# Survey-based socio-economic data from slums in Bangalore, India

**DOI:** 10.1038/sdata.2017.200

**Published:** 2018-01-09

**Authors:** Debraj Roy, Bharath Palavalli, Niveditha Menon, Robin King, Karin Pfeffer, Michael Lees, Peter M. A. Sloot

**Affiliations:** 1 University of Amsterdam, Amsterdam 1098 XH, The Netherlands; 2 Nanyang Technological University, Singapore 639798, Singapore; 3 Fields of View, Bangalore 560078, India; 4 Centre for Budget and Policy Studies, Bangalore 560004, India; 5 Urban Development, Ross Center for Sustainable Cities, World Resources Institute, Washington, DC 20002, USA; 6 School of Foreign Service, Georgetown University, Washington, DC 20057, USA; 7 National Research University ITMO, St Petersburg 197101, Russia

**Keywords:** Socioeconomic scenarios, Sociology

## Abstract

In 2010, an estimated 860 million people were living in slums worldwide, with around 60 million added to the slum population between 2000 and 2010. In 2011, 200 million people in urban Indian households were considered to live in slums. In order to address and create slum development programmes and poverty alleviation methods, it is necessary to understand the needs of these communities. Therefore, we require data with high granularity in the Indian context. Unfortunately, there is a paucity of highly granular data at the level of individual slums. We collected the data presented in this paper in partnership with the slum dwellers in order to overcome the challenges such as validity and efficacy of self reported data. Our survey of Bangalore covered 36 slums across the city. The slums were chosen based on stratification criteria, which included geographical location of the slum, whether the slum was resettled or rehabilitated, notification status of the slum, the size of the slum and the religious profile. This paper describes the relational model of the slum dataset, the variables in the dataset, the variables constructed for analysis and the issues identified with the dataset. The data collected includes around 267,894 data points spread over 242 questions for 1,107 households. The dataset can facilitate interdisciplinary research on spatial and temporal dynamics of urban poverty and well-being in the context of rapid urbanization of cities in developing countries.

## Background & Summary

Cities have become engines of accelerated growth as they are centres of high productivity and provide easy access to resources^1^. The outcome of this high rate of urbanization has been the rise of informal settlements or ‘slums’, characterized by a lack of adequate living space, insecure tenure and public services^2^. In 2010, an estimated 860 million people were living in slums worldwide with around 60 million added to the slum population between 2000 and 2010. In sub-Saharan Africa, the slum population doubles every 4.5 years^3^. In the past decade, over 22 million people have migrated from rural to urban areas in India^4^. While official estimates indicate that the number of slum dwellers in India increased from 30 million in 1981 to over 61 million in 2001^4^, a UN Habitat report estimates the number of slum dwellers in India to be over 100 million. In 2011, 200 million people in urban Indian households were considered to live in slums^5^, of which over a third were in million-plus cities of India.

In this paper, we present granular data about slums from the city of Bangalore in India. Bangalore grew exponentially from 1941 to 1971^6^ and is now rapidly growing due to the establishment of the software industry in the city. The urban agglomeration of Bangalore is the administrative capital of the state of Karnataka in India, with a metropolitan population of about 11.52 million and a population growth rate of 47.18%^5^, making it the third most populous city and fifth most populous urban agglomeration in India. The city has seen phases of growth that correspond to the different waves of industrialization and immigration. The first wave of immigration took place between 1880 and 1920, when the textile industry developed in the western part of the city. The second wave of industrialization took place in the eastern and northern areas, when a slew of state owned industries were created between 1940 and 1960. At the same time, state owned research and development establishments were created in the north western region of the city. The final wave can be characterized post 1990, with the establishment of special economic zones for electronics and the IT industry (which was initiated in the 1980s by the state government). To meet housing needs, in the period between the 1980s–1990s, state owned bodies created townships for their employees at the periphery, while housing co-operative societies met the demand for those in the formal sector^6^. A rapid shortage of housing and increased demand for manpower in the city has led to the growth and emergence of slums in Bangalore. The number of slums in Bangalore has grown from 159 in 1971 to over 2000 slums (notified and non-notified) in 2015. Those living in slums accounted for just over 10% of the city’s population in 1971 and an estimated 25 to 35% in 2015.

However, one of the biggest problems associated with studying slum populations is that, despite being ubiquitous, their needs, issues and problems are often invisible due to lack of representation. In this data collection effort the lack of accessibility to slums because of the social distance between the researcher and the respondent was overcome by the use of participatory methods. In order to measure poverty in slums, previous studies have often relied on consumption and income indicators. A Basic Needs Index requires data on literacy, water (piped), sanitation facilities and food requirements^7^. Well-being and vulnerability indicators have used household assets, access to financial services and formal safety nets and social networks^8^. In order to acknowledge the shift in thinking towards multi-dimensional poverty, we ensured that the survey moved away from consumption indicators to well-being and vulnerability indicators. The primary questions for this survey included the economic contribution of the urban poor in the city, the affordability and accessibility of infrastructure facilities, the various migration streams and access to financial systems. Using a participatory method, the survey was conducted in 1,107 households in 36 slums, with each household answering 242 questions. This study and the data descriptor provides a template for future data that can be collected to provide a better understanding of slums in other cities. The data can be used to generate a wide range of measures to study the impact of various programs on the slum dwellers, their expenditure patterns and the economic profile of the slums.

## Methods

The main purpose of the study was to obtain a better understanding of the nature of urban poverty, to unpack the needs, issues and problems of slum dwellers, but also how slum-dwellers contribute to the urban economy and why households live in slums. The primary research questions were:

- What is the economic contribution (labour, production aspects) of the urban poor to the city’s economy?
- What are the infrastructure facilities (health, education, water, mobility, sanitation) that are available? Are they affordable, accessible and who pays for it (state/private)?
- What are the key drivers of migration flows in and out of the city? When do people enter/leave a slum?
- What is the demographic and economic profile of the people living in slums?
- Do slum dwellers have access to financial systems and savings?
- What are the expenditures of people in the slums?

We combined a structured survey with focus groups and personal interviews. While the structured survey supported the systematic data collection, the use of the qualitative methods such as focus group discussions. Personal interviews allowed for individuals living in the slums to articulate their concerns and also supported further processing of the data, for instance to create categories. The design of the questionnaire was informed by our research questions and former surveys carried out in Bangalore, a survey developed earlier by the Word Bank^9^ and surveys reported in literature^10–12^. The slum-survey was done in collaboration with slum dwellers to get access to slum areas and have sufficient trust between surveyor and respondent to obtain a higher validity in the answers. The following sections describe the sampling strategy used to randomly select households and individuals for the survey and survey implementation.

### Sampling strategy

The slums in Bangalore were stratified based on the following parameters: Age of the Slum (Old, New), Location in the city (Core, Periphery, North, South, East, West); Size of the slum; Land Type (whether the slums are on Public land or Private land); Declaration Status (Declared or Not Declared); Major Linguistic Group (slums that contain a majority of Kannada, Tamil, or Telugu speakers); Major Religious Group (slums that contain a majority of Hindi, Muslim, or Christian populations) and State of Development (Redeveloped slums, Resettled slums, In situ developed and Planned slums). A list of 597 slums was compiled using the notified, non-notified and de-notified slums published by Karnataka Slum Clearance Board (now the Karnataka Slum Development Board). A total of 51 slums were short-listed based on the stratification criteria, after which 36 were surveyed based on verbal consent provided by the slum leaders. The following question guides the calculation of samples.

How many households should be surveyed to estimate the true proportion of households who are below the poverty line (or do not have access to finance/ water etc), with a 95% confidence interval 6% wide? The 95% confidence interval is a standard used across disciplines. We have used a width of 6% instead of 10% (standard used across disciplines) to overestimate the number of samples (in case of missed households).

The required sample size (*X*) for the slum households was then calculated using the following formula^13^:

(1)X=n1+(n/N)

where, *N* is the entire population of slum households in Bangalore and

(2)n=Z2D2[P×Q]

where *Z* is the Z-score (*Z* is 1.96 for a 95% confidence level), *D* is the margin of error (3%), *P* is the estimated proportion of an attribute (such as households below poverty line) that is present in the population, *Q* is 1−*P*. Therefore, *P*×*Q* is the estimate of variance of the attribute in the population. Because a proportion of 0.5 indicates the maximum variability of an attribute in a population, it is often used in determining a more conservative sample size. Therefore, applying equation (2), we get *n*=1,067. The total number of slum households in Bangalore was estimated to be 321,296 (N ) as per the report released by the Karnataka Slum Development Board in 2010. Since, N≫*n*, in equation (1), the calculated sample size is 1,067.

### Survey implementation

The social survey was implemented in the city of Bangalore, India with the assistance of Fields of View (FoV), a non-profit research organization, highly experienced in data collection. FoV together with Slum Jagaththu provided intensive training on survey tools, data collection methodology and ethical grounds of social data collection. The questionnaire was designed based on the research questions, after which the stratification and identification of slums (described in section Sampling Strategy) was performed. After the slums were identified, the questionnaire was modified to include questions based on the input and needs of the slum dwellers (see Table 1). A set of qualitative interviews on thematic topics were carried out based on the request from the slum dwellers, which served as a reference point for comparison with past surveys. The questionnaire was piloted in 2 slums and then revised after a round of feedback (see Table 2). A typical survey procedure consisted of the field coordinator speaking to the local leaders in each slum before the team conducted the survey. The coordinator would then introduce the enumerator team covering that slum to the slum leaders. If the slum leaders were agreeable, they would survey 10% of the slum, based on the procedure elaborated above. Informed consent was obtained at 3 different levels. First, local slum leaders were apprised of the objectives of the survey and the methods. Once local slum leaders approved the survey, we identified and approached community leaders within a slum. After the consent of the community leaders was obtained we approached individual slum households. Efforts were made to ensure that all respondents were appropriately informed about the study and thoroughly understood their participation in the study was voluntary. In the cases where slum leaders or community leaders did not agree to the survey we did not proceed further. In all cases where community leaders agreed to be a part of the survey, all households complied to the request. Participation was voluntary and interviewers ensured that participants knew that refusal to participate would not lead to any adverse consequences. If the main earner was not available at the time of interview then the enumerator excluded that household and moved to the next selected household and then reverted to the normal pattern.

The data was captured in paper questionnaires with handwritten responses, with most answers coded into structured replies (as indicated in the validation section), in addition to a few open-ended questions. Several case studies on thematic topics such as the homeless, informal workers and street vendors were also conducted. These case studies were conducted based on qualitative interviews with the participants and the data is not included in the datasets. The survey was administered by women participants from the slums in order to increase the level of comfort and trust with the participants. The enumerators comprised of fourteen women, who conducted the surveys in teams of two. The questionnaire was developed in English and then translated into Kannada (the local language of the state of Karnataka). To ensure quality of the data, a monitoring team from FoV checked one percent of the data and held periodic meetings to provide necessary feedback regarding the field work. The survey was completed in two stages, the first beginning in June 2010 included 20 slums and the second, beginning in March 2011 included 16 slums (see Table 3). Direct observation or spot checking in selected houses and re-interviewing with a quality control questionnaire in selected households formed part of the monitoring process. Survey data and accompanying questionnaires are available on the ReShare Repository (Data Citation 1).

The data collected from this survey underwent cleaning and was stored in a relational database for further analysis. More specifically, the data was vetted by the enumerators and research team by randomly picking households and a site visit with field verification was carried out. Once the data was verified by the surveyors, the filled-in questionnaires were translated to English and then digitized by an independent group. The research team then carried out two rounds of validation, in the first round, the data was checked for consistency and outliers and in the second round, the research team coordinated with the enumerators to validate any discrepancies. This paper describes the relational model of the database, how to use it, the variables in the dataset, the variables constructed for analysis and the issues identified with the dataset. The data collected included 267,894 data points spread over 242 questions for 1,107 households.

### Code availability

This study did not use any computer codes to generate the dataset. A MySQL relational database was used to store the collected data. A set of SQL queries were used to verify and validate the data.

## Data Records

The Survey data is provided in SQL format (Data Citation 1). All 242 questionnaire variables are named according to their number in the questionnaire and fully described in the variable labels. The household listing and survey instruments can be downloaded in English which acts as the code book for the datasets (Supplementary File 1).

## Technical Validation

The technical validation and quality control comprised of three stages. The first stage of quality control was done before the survey was carried and it involved: a) thorough pre-testing of the questionnaire; b) translating the questionnaire into Kannada, including local terminology and reverse translating to check quality of translations; c) recruitment of women enumerators from slums and comprehensive training in survey implementation. The survey questionnaire was designed based on the research questions of the project, using questions from other surveys already implemented in India and drawing on the qualitative data collection and expert judgement to create new questions. To ensure that the questions are relevant and meaningful, pre-testing of the quantitative questionnaire was conducted in the study area through pilot surveys and focus group discussions (described in Survey Implementation) prior to finalisation of the questions. Training of the enumerators is essential for effective implementation of a survey. A deep understanding of the questions and philosophy of the survey ensured that enumerators can help the surveyed households in answering the questions properly. To achieve this, the enumerator team was selected from the local slums (described in Survey Implementation). Role play and field practice was carried out for every section of the questionnaire.

The second stage of validation was performed during the survey and it involved field quality control questionnaires being carried out alongside the main data collection as described below. A quality control team was assigned in the field to monitor data collection. The field quality control involved quality control visits, spot check visits and checking of forms as recommended by the Demographic Surveillance Systems guidelines. Quality control visits was done by the supervisor on 5%^14,15^ of the households in each round of data collection. It provided a way of cross-checking the accuracy and completeness of the data. Random and unannounced spot check visits were conducted to ensure that the data collection was being done as per the schedule. Finally, during the survey a field supervisor checked all the completed forms for completeness (no missing values and units) and accuracy before they were submitted for data entry. To ensure the data is accurate all possible inconsistencies (e.g., range checks, checking that only females have given birth) were checked. Forms with omissions and obvious errors were returned to the fieldworker for correction or revisits. The field quality control exercise demonstrated that most respondents were not willing to disclose their caste as it is considered sensitive in India. At the point of data entry a further checking of the forms is performed and forms that have errors or inconsistencies were returned to the fieldworker via his/her supervisor. The built-in validation during data entry comprised of standard methods such as uniqueness check, referential integrity, presence check, length check, data type check, fixed value check and cross field check^16^.

The final stage of validation was after the survey was completed and it involved checking data entry, detecting typing errors and comparison with previous studies. Two-pass verification, also called double data entry was performed to ensure correct data entry. To identify data entry errors, individual and composite variables were summarised as minimum, median, mean, maximum and compared between the two data entries. The original paper version has been retained to allow the team to check individual records in the digital dataset if necessary. Further, in this section we present a detailed quantitative validation of the survey data by comparing frequency distributions with previous studies and census surveys. First, we validate the demographic variables in the survey.

**Figure 1:**
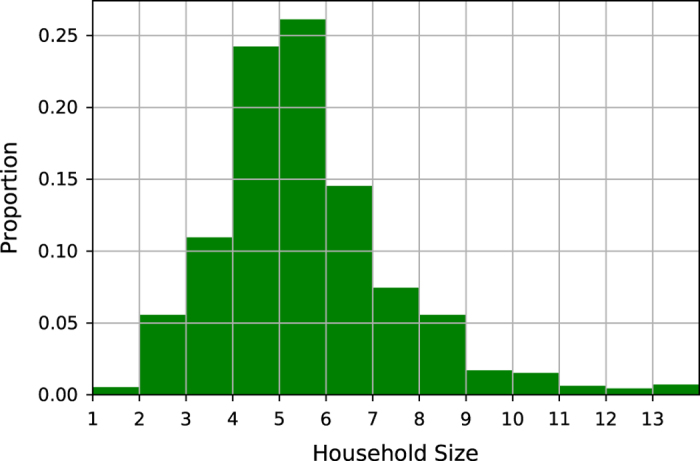
Household Size Distribution across the 36 slums in Bangalore.

The median household size in the slums of Bangalore is 5. We find that 25% of the families have a household size of up to 4 members and 75% of the slum dwellers have a household size of up to 6 members. The maximum size of a household in the survey is 13. Figure 1 shows the family size distribution across the 36 surveyed slums in Bangalore. Table 4 indicates that the gender ratio (female to male ratio) is around 1, which is different to the trend in non-slum urban households where there are around 966 female per 1,000 male. A similar deviation has been observed in the Census of India 2011^5^ and other slum studies in Bangalore^17^. Table 4 also shows that the population in the slum is young, with 35% of the respondents under the age of 18 and around 70% under the age of 35. The age distribution is consistent with the data from Census of India 2011^5^. The majority of surveyed households (67%) are Hindus. About 20% of the respondents are Muslims and 8% are Christians. The native language of 45% of surveyed households is Tamil, while 17% speak Kannada and 15% speak Telugu. Analysis of the migration data from slums show that 73% of migrants are from rural areas within Karnataka itself while the remaining 27% migrated from the rest of India, which indicates that the native language may not be an indication of migration. The above social and demographic distribution are similar to values reported in various slum studies of Bangalore^17–21^.

The data indicates that the average age at marriage is 24 for men and 17 for women. This is lower than the average of non-slum urban households in Bangalore, where the average age of marriage is 27.5 for men and 24 for women^5^. The median age of marriage has been rising in India. However, 49% of all women in the survey, were married before the age of 17. The median age at first pregnancy in slums of Bangalore is around 18 years, which is significantly lower than the median age of 25 years for non-slum urban households in Bangalore^5^. The average age at marriage and pregnancy are similar to the reported values in various slum studies of Bangalore^22,23^.

**Figure 2:**
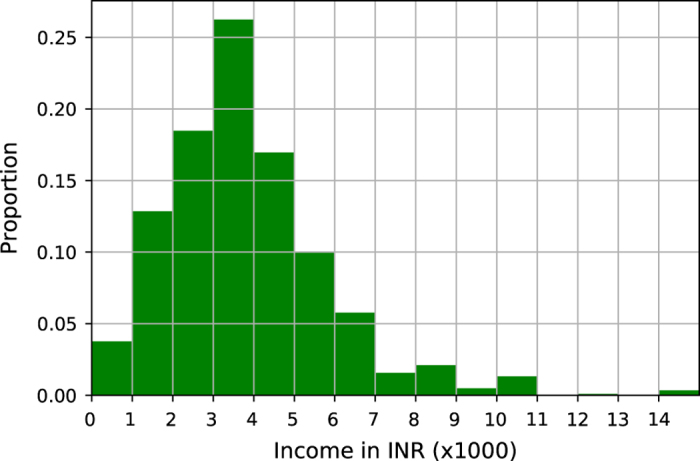
Income Distribution of Slum Dwellers in the 36 slums of Bangalore.

Second, we validate the data pertaining to income, expenditure and assets in the survey. The income distribution (see Fig. 2) shows that 25% of sample respondents earn a monthly income of less than 2,000 INR (31 USD), out of which they spend 93% on basic amenities. Around 75% of the sample respondents earn a monthly income lower than INR 4,000 (62 USD) and spend 91% of their income on basic amenities. Around 13% of households earn more than 10,000 INR (156 USD) per month and spend around 77% of their income on basic amenities. The monthly median income of slum dwellers in Bangalore is around 3,000 INR (47 USD). The median income reported in previous studies is around 3,500 INR (54 USD)^17–19^. Table 5 shows that the slum households spend the majority of their income on food items. The reasons for this high percentage are low income level coupled with high food inflation based on the Consumer Price Index (CPI) which was 12.56% during 2013. Hence, money available for other activities is very low. Education is a priority for the urban poor, but only the top 10% highest earning households can afford a school education for their children. The other key components which contribute to the expenditure are home appliances, rent, healthcare and clothing. The expenditure patterns observed in the data are consistent with previous findings^17,18^.

In the surveyed slums, television sets, mobile phones and electric fans are the common asset types, with more than 75% reporting ownership of each of these assets. The least common form of assets are cars, trucks and agricultural land, possessed by less than 1% of all slum households. Bicycles or motorcycles/scooters are also owned by fewer than 20% of these households. The asset distribution we observe is similar to previous studies in the slums of Bangalore^17,18^.

When we examine the employment patterns in the slums, we find that most slum dwellers are employed in the informal sector, primarily working as domestic help or as manual labour. Only 13% of the sample respondents are employed in the formal sector (White collar, blue collar and sales occupations). These findings are similar to the reported values in various slum studies of Bangalore^17–21^. Further, we find that the slum-based micro enterprises are not served by traditional financial institutions due to their informal status (Table 6). Again, this is consistent with previous studies, for example the study conducted by Society for Participatory Research in Asia^17^.

Finally, we validate the data pertaining to physical structure of the houses and tenure in the survey. The survey data indicates that around 40% of the slum households have *Hakku Patra*, which is an important document given by *Tehsildar* for land ownership indicating title to the dwelling. This indicates that the majority of slum dwellers possess legal titles. Households with a legal title to their dwelling usually live in *pukka* structures. A *pukka* structure is a semi-permanent structure with a tiled or stone roof and walls that are wooden, metal, asbestos sheets, burnt brick, stone, concrete or cement bricks. Around 20% of the households have a Possession Certificate document and live in semi-pukka houses. The remaining 40%, who have either migrated from neighbouring districts or other states, do not have any proper ownership to land and live in *kutcha* structures. A *kutcha* structure is one whose roof is built using grass, thatch, bamboo, plastic, polythene, metal, asbestos sheets and walls that are grass, thatch, bamboo, plastic, polythene, mud, burnt brick, wood, metal, asbestos sheets (See Table 7). Analysis of ration card data from the slums in Bangalore shows that around 3% of households have *Antyodaya* cards, 60% possess below poverty line (BPL) cards and 17% have the above poverty line (APL) card. A comparison with the study conducted by Society for Participatory Research in Asia^17^ shows that the above distributions are comparable.

## Usage Notes

### Data access conditions

A benefit of the data is its spatial nature, which allows social factors to be analysed in the context of environmental conditions and resources. However, this increases the sensitivity of the data as it creates the potential for households within each slum to be identified from the survey data. As such, the data has been made available as safeguarded on the UK Data Archive’s data repository ReShare. In order to download safeguarded data the user must register with the UKDA and agree to the conditions of their End User Licence (For conditions of the End User Licence see: https://www.ukdataservice.ac.uk/get-data/how-to-access/conditions). For commercial use, please contact the UK Data Service at help@ukdataservice.ac.uk.

The diversity of variables collected in the survey instrument create a high possibility for reuse of this dataset. Furthermore, certain variables are comparable with the standard National Family Health Survey v.2,3,4 surveys of Bangalore and the national census, offering the possibility of longitudinal analysis. The dataset can be used to test key associations between social and land-use outcomes that are critical for environmental policy and development strategies for Bangalore.

For example, there are a range of variables that will allow researchers to examine the social relationships that affect livelihoods in slums such as money lending, informal labour, remittances and assets. Comprehensive data on expenditure, income and livelihood choices could be used to model growth and emergence of slums (e.g., ref. 24) and design strategic slum management interventions, ranging from improvements in public distribution system, through to social interventions in availability of credit, or supporting mobility and migration. The dataset can be disaggregated by group identities, and crucially includes information on seasonal variation in occupation and livelihoods, a critical issue in the variation of well-being and poverty.

## Additional information

**How to cite this article:** Roy, D. *et al.* Survey-based socio-economic data from slums in Bangalore, India. *Sci. Data* 5:170200 doi: 10.1038/sdata.2017.200 (2018).

**Publisher’s note:** Springer Nature remains neutral with regard to jurisdictional claims in published maps and institutional affiliations.

## Supplementary Material



Supplementary File 1

## Figures and Tables

**Table 1:** Minor modifications of questionnaire based on the interests of slum dwellers.

**S. No.**	**Theme**	**Feedback**	**Modification**
1	Education	Ability to read and write	Add a sub-question on ability to read and write
2	Employment	Seasonality of jobs	Modified questionnaire to add seasonality to nature of job
3	Migration	Movement within Bangalore	Added questions on reasons for movement within different slums
4	Mobility	Different options used for different needs	Sub-divided questions to work, education, household purchases
5	Water	Multiple sources of water	Added section on sources of water
6	Borrowing for land	Borrowing through multiple sources	Added questions on specific sources and details on interest rate, term and EMI
7	Assets	Assets were mostly second hand	Question modified to include price paid at the time of acquisition

**Table 2:** Minor modifications of questionnaire after pilot survey.

**S. No.**	**Theme**	**Feedback**	**Modification**
1	Education	Attending informal school or training	Added non-formal schooling
2	Employment	Employee benefits or welfare	Added question on benefits and welfare
3	Self Employment	We spend our own resources	Questions added for instruments used, capital invested and the source of the capital.
4	Transportation	Information about transport	Changes made to ensure that information sources are captured
5	Water	Payment mode and collectivisation	Added questions on to whom was the payment made for water and on how private modes of water is bargained for and delivered.
6	Issues, Agency Benefits	There are some people who help with problems	Questions modified on organisation working on welfare of slums and actors who interact with the slum dwellers

**Table 3:** Two rounds of survey were implemented between June 2010 and March 2011.

**Round**	**Action**	**Comment**
1	Surveyed 20 slums	Slums were surveyed and households unavailable (comment 3) were marked. They were then revisited in the second round.
2	Surveyed remaining 16 slums	This breakdown was based on availability of the survey team and access to the slums. Households that were unavailable (comment 3), were revisited during the spot-checking phase to collect the data.

**Table 4:** Gender and Age Distribution across the 36 slums in Bangalore.

**Demographic Variable**	**Classification**	**Proportion of Respondents**
Gender	Male	49.01
	Female	49.35
	Transgender	0.19
Age	Under 18	34.93
	18–24	17.64
	25–34	17.89
	34–60	25.34
	60 above	4.19

**Table 5:** Comparison of CPI between slums and non-slum households.

**Groups**	**Bangalore Urban**	**Bangalore Slums**
Food & Water	38.26	55.91
Fuel	8.82	3.12
Housing	26.04	7.23
Clothing	2.59	0.59
Education	2.37	1.11
Health Care	3.45	7.21
Entertainment	2.10	2.34
Transportation	9.56	11.21
Source:Central Statistics Office, February 11, 2014.		

**Table 6:** Sources of Finance for Self-Employment.

**Source**	**Percentage**
1. Own sources	37.29
2. Informal borrowing(no interest)	7.63
3. Informal loan or money lender	46.61
4. Formal loan(without collateral)	5.08
6. Other	3.39

**Table 7:** Housing types based on construction material.

**Housing Type**	**Percentage**
Kutcha	40.50
Semi-pucca	19.74
Pucca	39.75
