# Supplementary material for: Survey-based socio-economic data from slums in Bangalore, India
Source: Sci Data. 2018 Jan 9;5:170200. doi: 10.1038/sdata.2017.200 (PMC5759370; doi:10.1038/sdata.2017.200)
Supplement: Supplementary File 1 [file sdata2017200-s2.pdf]

Supplementary File 1

User Manual

For use with the survey data of the Urban Poverty Study, 2010

November 14, 2017

Fields of View

Contents

1 Introduction **1**

2 Setup of the database **2**

3 Database Structure and Usage **3**
- 3.1 Naming Convention 3
- 3.2 Enhanced Entity-Relationship diagrams 5

4 Construction of variables **18**
- 4.1 Family Categories 18
  - 4.1.1 First version of the categorisation 18
  - 4.1.2 Second version of the categorisation 19
- 4.2 Occupation Categories 20
- 4.3 Housing 26
- 4.4 Income Categorisation - Individual Income 27
  - 4.4.1 First version of the categorisation 28
  - 4.4.2 Second version of the categorisation 29
- 4.5 Income Categorisation - Household Income 30
- 4.6 Age Groups 32
- 4.7 Age of the slums 34

5 Issues and Resolution **39**

References **44**

Appendices **45**

A Ration Card types **46**
- A.1 Akshaya Scheme (Yellow Card) 46
- A.2 Anthyodaya Anna Yojane 47
- A.3 APL card 47

B EER Diagram Guide **48**

C Sample SQL Queries **50**

D Variables **52**

List of Figures

3.1 Household 6
3.2 Employment 7
3.3 Migration 8
3.4 Travel 9
3.5 Water (Questions 91 to 108) 10
3.6 Water (Questions 109 to 129) 11
3.7 Water (Questions 130 to 143) 12
3.8 Housing (Questions 144 to 155) 13
3.9 Housing (Questions 156 to 171) 14
3.10 Housing (Questions 172 to 189) 15
3.11 Income, Expenditure and Assets 16
3.12 Issues, Agencies and Benefits 17

4.1 Income Distribution in the data 28
4.2 Frequency Distribution for Income Intervel-Set 1 29
4.3 Frequency Distribution for Income Intervel-Set 2 29
4.4 Frequency Distribution for Income Intervel-Set 3 30
4.5 Frequency Distribution for Income Intervel - Final 30
4.6 Household Income Distribution in the data 31
4.7 Frequency Distribution for Income Intervel-Set 1 31
4.8 Frequency Distribution for Income Intervel-Set 2 32
4.9 Frequency Distribution of age groups, Intervel-Set 1 33
4.10 Frequency Distribution of age groups, Intervel-Set 2, for literacy data analysis 33
4.11 Frequency Distribution of age groups, Intervel-Set 2, for occupation data analysis 34

B.1 Sample EER diagram 48

List of Tables

4.1 Occupation Categories - version 1 21
4.2 Occupation Categories - Final version 24
4.3 Age of the slums, version 1 34
4.4 Age of the slums as identified from case studies 36
4.5 Age of the slums as per KSCB surveys 36
4.6 Age of the slums, final version 37

5.1 Issues with the current dataset 40

A.1 Facilities available through a Yellow Card 47
A.2 Facilities available through an Anthyodaya Anna Yojane card 47
A.3 Facilities available through an Anthyodaya Anna Yojane card 47

D.1 Household and demographics 53
D.2 Employment 53
D.3 Migration 54
D.4 Travel 56
D.5 Water 58
D.6 Housing 61
D.7 Housing Finance 62
D.8 Income and Expenditure 64
D.9 Remittances and Loans 64
D.10 Household Assets 66
D.11 Issues, Agencies and Benefits 66
D.12 Code tables in the database 68
D.12 Code tables in the database 69

1 | Introduction

The survey of Bangalore slums carried out in the year 2010 covered **36 slums** across Bangalore. The slums in Bangalore were stratified based on the following parameters: Age of the Slum (Old,New), Location in the city (Core, Periphery, North, South, East, West); Size of the slum; Land Type (whether the slums are on Public land or Private land); Declaration Status (Declared or Not Declared); Major Linguistic Group (slums that contain a majority of Kannada, Tamil, or Telugu speakers); Major Religious Group (slums that contain a majority of Hindi, Muslim, or Christian populations) and State of Development (Redeveloped slums, Resettled slums, In situ developed and planned slums). In order to ensure the quality of data, we short-listed 36 slums (based on access to the slums) that satisfied the criteria listed above. In this study, we decided to have a 10% sample size within each slum, i.e every 10th house, working from the street layout of the slum was part of the survey (as a formal list of households was unavailable).

A total of **1107 households** was surveyed. Each survey had **242 questions** (see attached questionnaire) spread across the following themes: demographics, employment, migration, transport, water, housing, loans, health-care, assets, aspirations, and problems.

This survey was part of the Bangalore Urban Poverty Study conducted in 2010 by multiple partners, and available through a Creative Commons license at: `http://ngil.cstep.in/projects/urban_poverty_survey.html` (Last accessed on 30 September 2013). Key assistance was provided by Mr. Issac Arul Selva and Mr. Issac Amruthraj. This survey was partly funded by the Jamsetji Tata Trust and the Next Generation Infrastructure Foundation.

The verification, authentication, structuring and analysis of this data, and this report on living wages was carried out by Fields of View in collaboration with the International Institute of Information Technology - Bangalore, and was funded by the University of Amsterdam.

The data collected from this survey underwent extensive cleaning, and was stored in a relational database for further analysis. This document describes the relational model of the database, how to use it, the variables in the dataset, the variables constructed for analysis and the issues identified with the dataset.

Chapter 3 desribes the database structure with instructions on using it, and the Enhanced Entity-Relationships models. Chapter 4 describes the additional variables that were constructed for analysis. Chapter 5 describes the documented issues with the data, and their resolution.

Appendix A provides details about the different ration cards provided by the Government of Karnataka. Appendix B provides a guide to reading the EER diagrams. Appendix D describes all the variables in the dataset.

2 | Setup of the database

The data is packaged as a MySQL .sql file, which is to be extracted into the MySQL server before use [1].

The minimum requirements for setting up this database are:

- A computer running either Linux, Windows or Mac
- MySQL server version 5.5+
- MySQL client version 14+, distribution 5.5+

This chapter describes the instructions to install the database on a typical computer where the MySQL server and client are running on the same computer.

The following steps are to be followed to set-up the database:

1. Login to MySQL server:

   ```
   $ mysql -u <rootusername> -p<rootpassword>
   ```

2. Create a database:

   ```
   mysql> create database urbanpoverty2010
   ```

3. Create a user and grant access: Consider the username to be urbanpovertydatauser and password as urbanpovertydatapwd

   ```
   mysql> grant all on urbanpoverty2010.* to
   >'urbanpovertydatauser'@'localhost'
   >identified by 'urbanpovertydatapwd';
   ```

4. Logout of MySQL by pressing Ctrl+C

   ```
   mysql> quit
   ```

5. Create the tables and populate the data with the following command:

   ```
   $ mysql -u urbanpovertydatauser -purbanpovertydatapwd
   mysql> use urbanpoverty2010
   mysql> source urbanpoverty2010.sql
   ```

   This will create the necessary tables and populate it with the data.

[1]It is assumed that the reader is well acquainted with the basics of MySQL

3 | Database Structure and Usage

The data collected included **242 questions** for **1107 households**. This data needed to be stored in a manner which was easily accessible, while maintaining the relationships between the data points.

For data integrity, ease of access, and for the ability to "pull" the data into a software/ tool of the users' choice for analyses, a Relational Model of data storage was chosen. The rationale for choosing a relational model is explained in more detail in the appendix. The entities and relationships within the Relational Model were designed such that the model resembled the survey instrument, while maintaining data integrity. The naming convention, described in Section 3.1, was designed so that people familiar with the survey instrument could easily identify the Relation (the RDBMS table) in which the corresponding data could be found.

3.1 Naming Convention

The database follows a standard naming convention across all its tables and columns.

1. **Table names**

   (a) For tables which store data about multiple questions, the naming convention is
   <questionnumber_from>_<questionnumber_to> _ThemeOfTheTable
   For example:

      i. The table for basic demographics of the household is called 1_5_Household as it contains the responses for questions 1 to 5, and is about the Household.

      ii. The table for details about people is called 8_15_Person as it contains the responses for questions 8 to 15.

   (b) For tables which store data about one question, the naming convention is
   <questionnumber>_ThemeOfTheTable
   For example:

i. The table for the material of the roof for the household is called 148_RoofMaterial as it contains the responses to just question number 148.

2. **Column names**

   (a) For columns which store data about one question, the naming convention is *<questionnumber>_questionDescription*
   For example

   i. The column 1_type_of_household in the table 1_5_Household contains the responses for the first question, Type of household

   ii. The column 11_age in the table 8_15_Person contains the responses for question number 11, What is the age of the person?

   (b) For columns which store similar data but about multiple questions, the naming convention is
   <questionnumber_from>_<questionnumber_to> _questionDescription
   For example, the column 61_66_71_cost stores the travel cost for travel to work (question number 61), travel to education (question number 66) and travel fr household purposes (question number 71). In the table, these are differentiated by the column “type_of_travel”, which could be 1 for work, 2 for education or 3 for household purposes [1].

3. **Code tables**: The table names for the code tables follow the following naming convention
   <NameOfTheCode>.
   The column names in these code tables follow the following naming convention *<name_of_the_code>_id* and *<name_of_the_code>_name*.
   For example, the table “TravelMode” stores the various travel modes people use. The column names are “travel_mode_id” and “travel_mode_name” which store the unique id of the mode and the name of the travel mode (Bus, Car, Two wheeler, etc.) respectively.

Certain accepted standard RDBMS naming conventions were followed. Table names are in singular (for example 8_15_Person, and not 8_15_People). Column names are not camel case, but underscore separated (18_occupation_id and not 18_occupationId).

However, table names are camel cased to distinguish them from column names, and for readability. All tables are normalized upto the third normal form (Elmasri & Navathe, 2008).

[1] The data collected for travel for household purposes is not cleaned due to multiple discrepancies in the data. This is documented in Section 5

3.2 Enhanced Entity-Relationship diagrams

The database was designed by modelling the data using Enhanced Entity- Relationship (EER) models (Elmasri & Navathe, 2008). The first version was designed using Aquafold Data Studio [2] for PostgreSQL [3], and the consequent versions were designed for MySQL [4] using MySQL Workbench [5].

This chapter provides the EER diagrams for this database. Since the database has 260 tables, these tables have been arranged theme-wise.

Figure 3.1 is the EER model for the tables relating to the household demographics data. Figure 3.2 is the EER model for employment data, Figure 3.3 for the migration data and Figure 3.4 for the travel data. Figures 3.5, 3.6 and 3.7 are the EER models for the data relating to water supply, cost and availbility. Figures 3.8, 3.9 and 3.10 are the EER models for the housing data. Figure 3.11 is the EER model for income, expenditure and assets. Figure 3.12 is the EER model for the qualitative data on issues, agencies and benefits.

Appendix B provides a guide for reading the EER diagrams. For a detailed explanation of relational database design and EER diagrams, please refer Elmasri et. al (Elmasri & Navathe, 2008) and Ramakrishnan et. al (Ramakrishnan & Gehrke, 2000).

[2]Aquafold Data Studio, http://www.aquafold.com/. Last Accessed on 24 September 2013

[3]PostgreSQL, http://www.postgresql.org/, Last Accessed on 24 September 2013

[4]MySQL, http://www.mysql.com/, Last Accessed on 24 September 2013

[5]MySQL Workbench, http://www.mysql.com/products/workbench/, Last Accessed on 24 September 2013

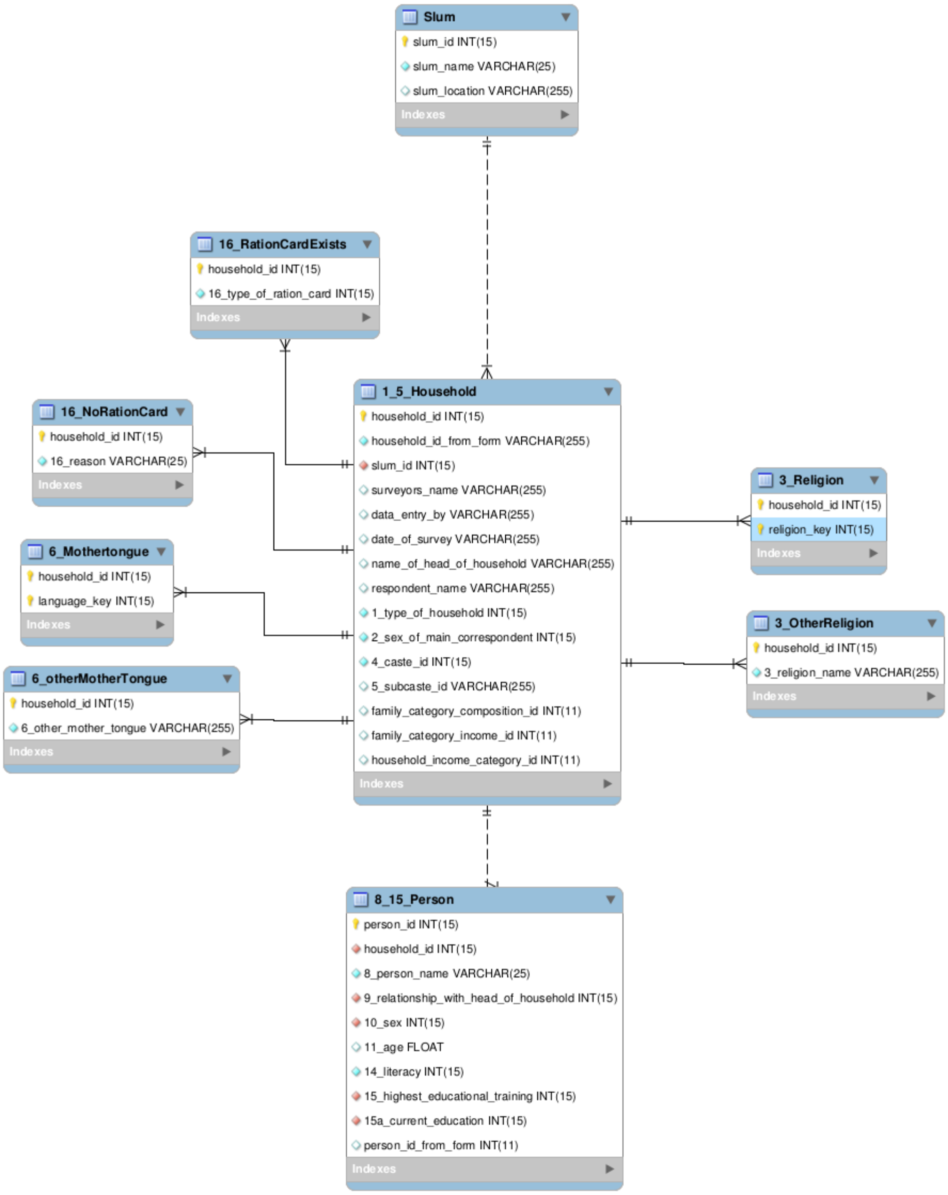

Figure 3.1: Household

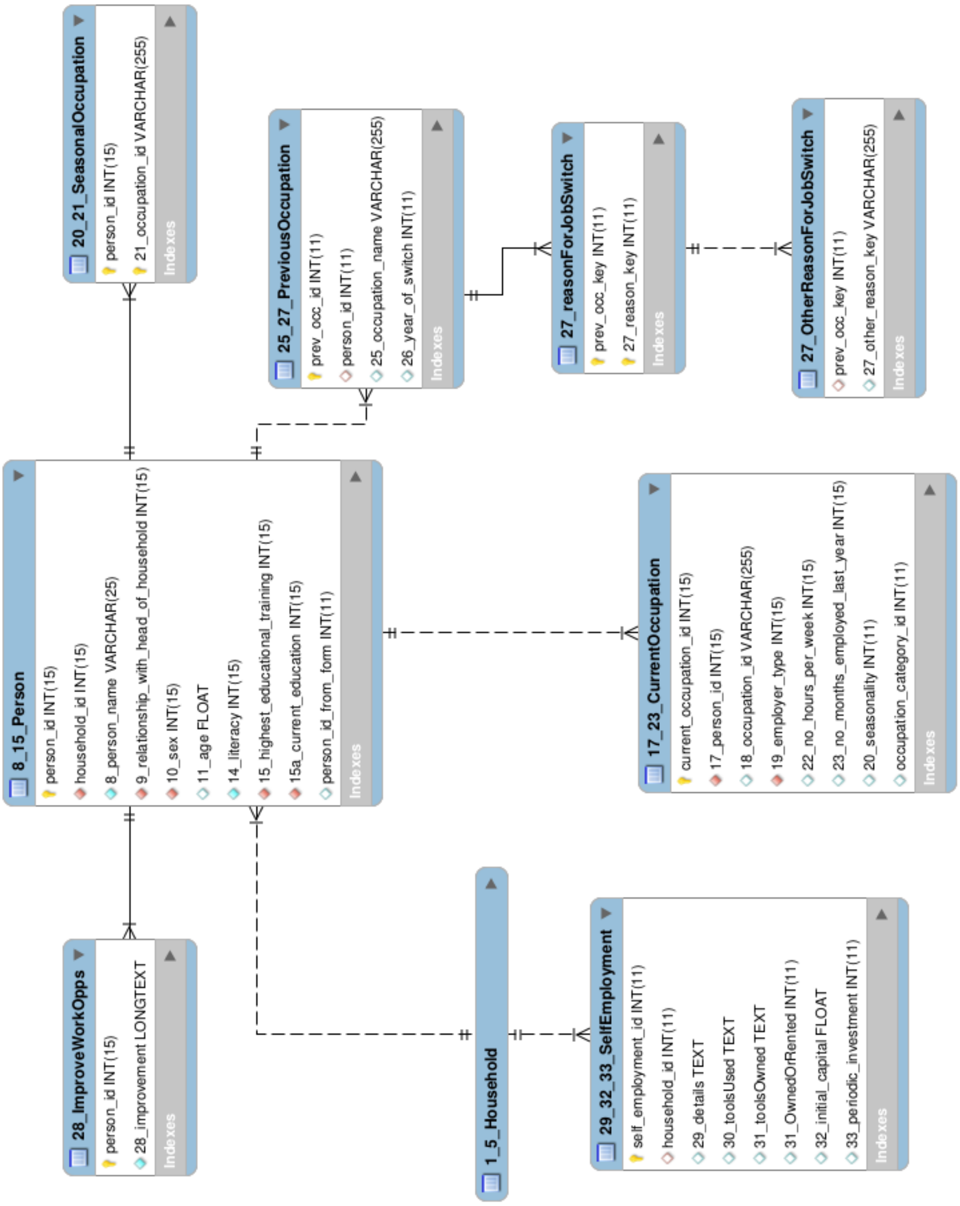

Figure 3.2: Employment

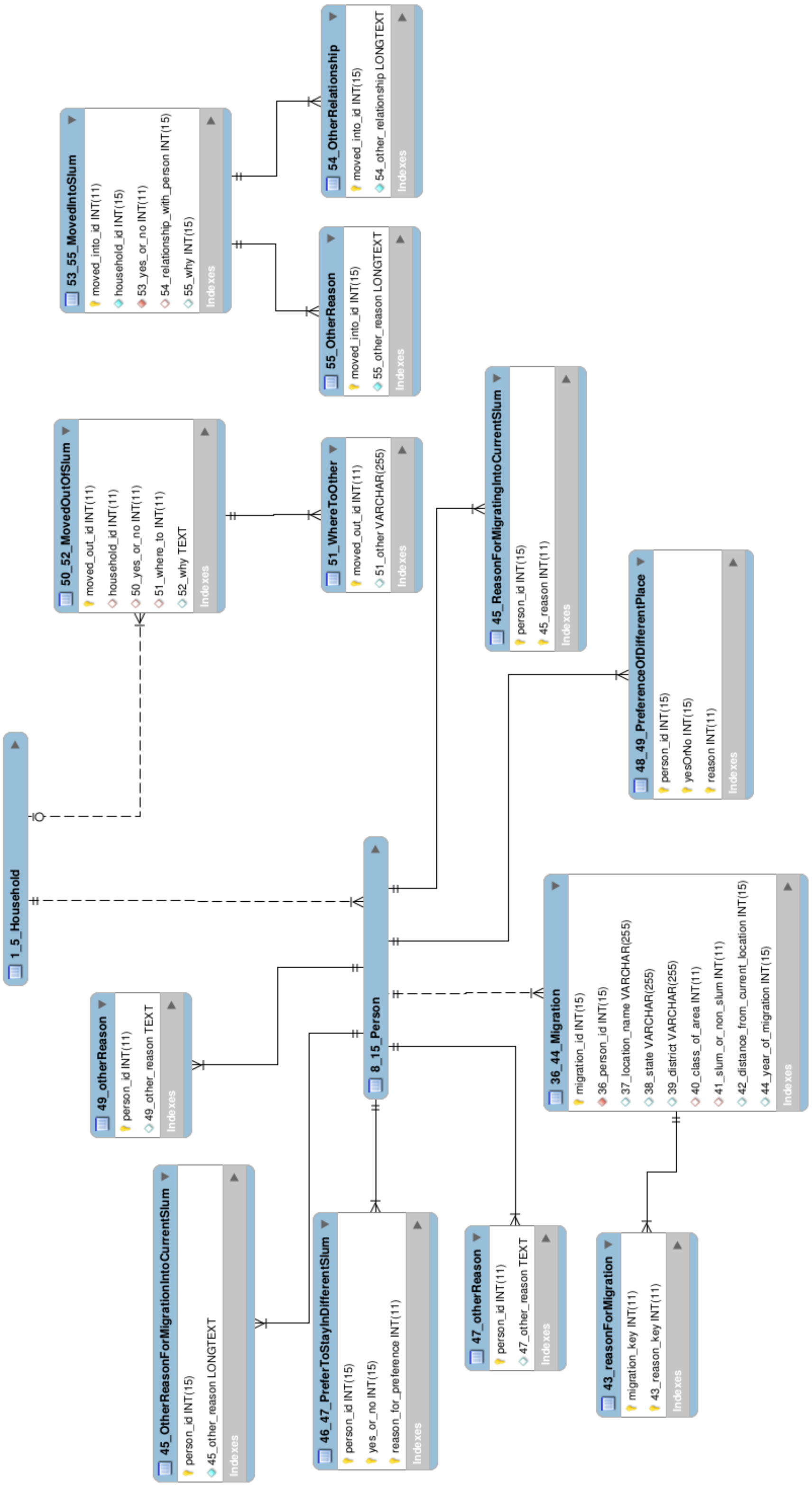

Figure 3.3: Migration

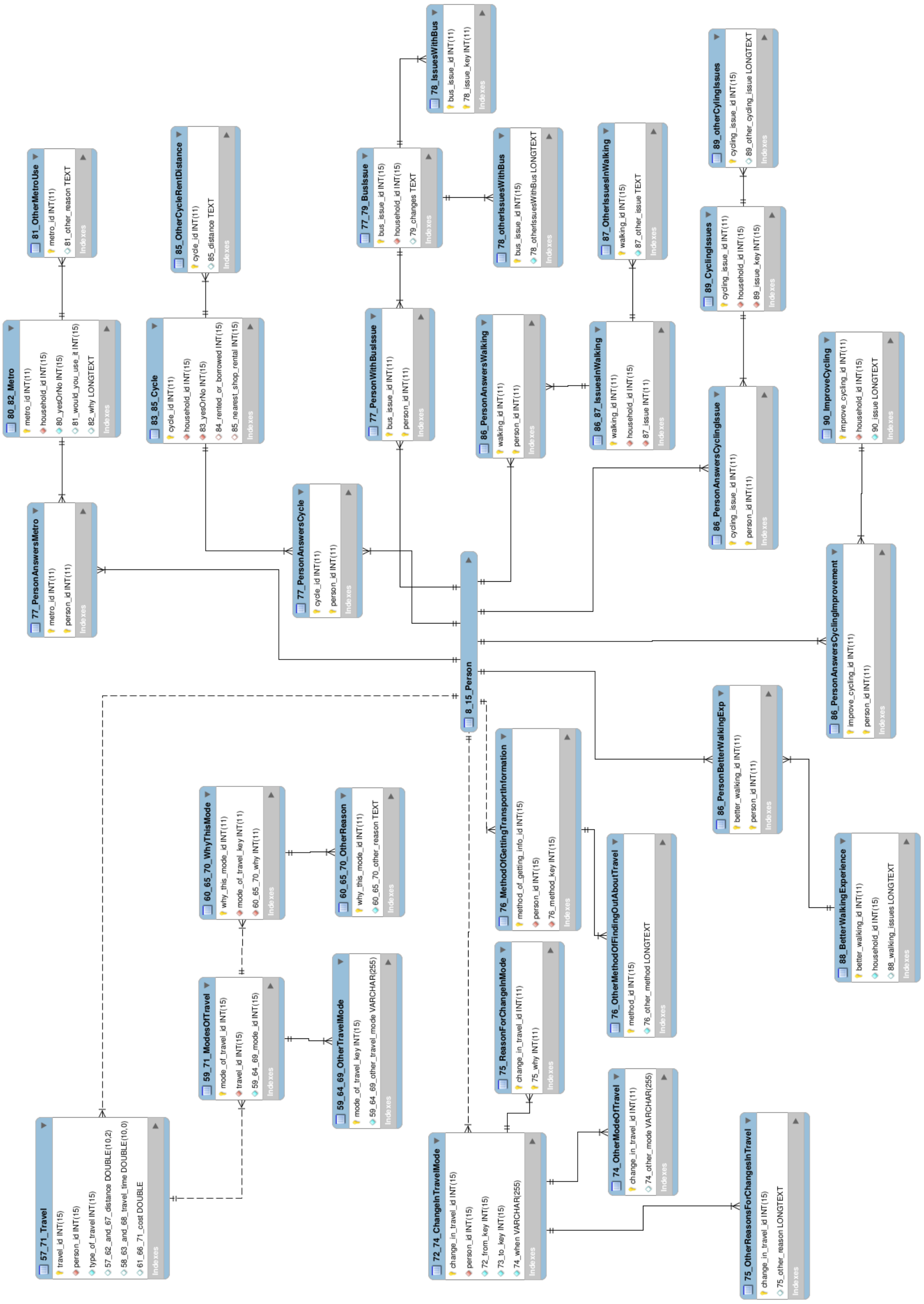

Figure 3.4: Travel

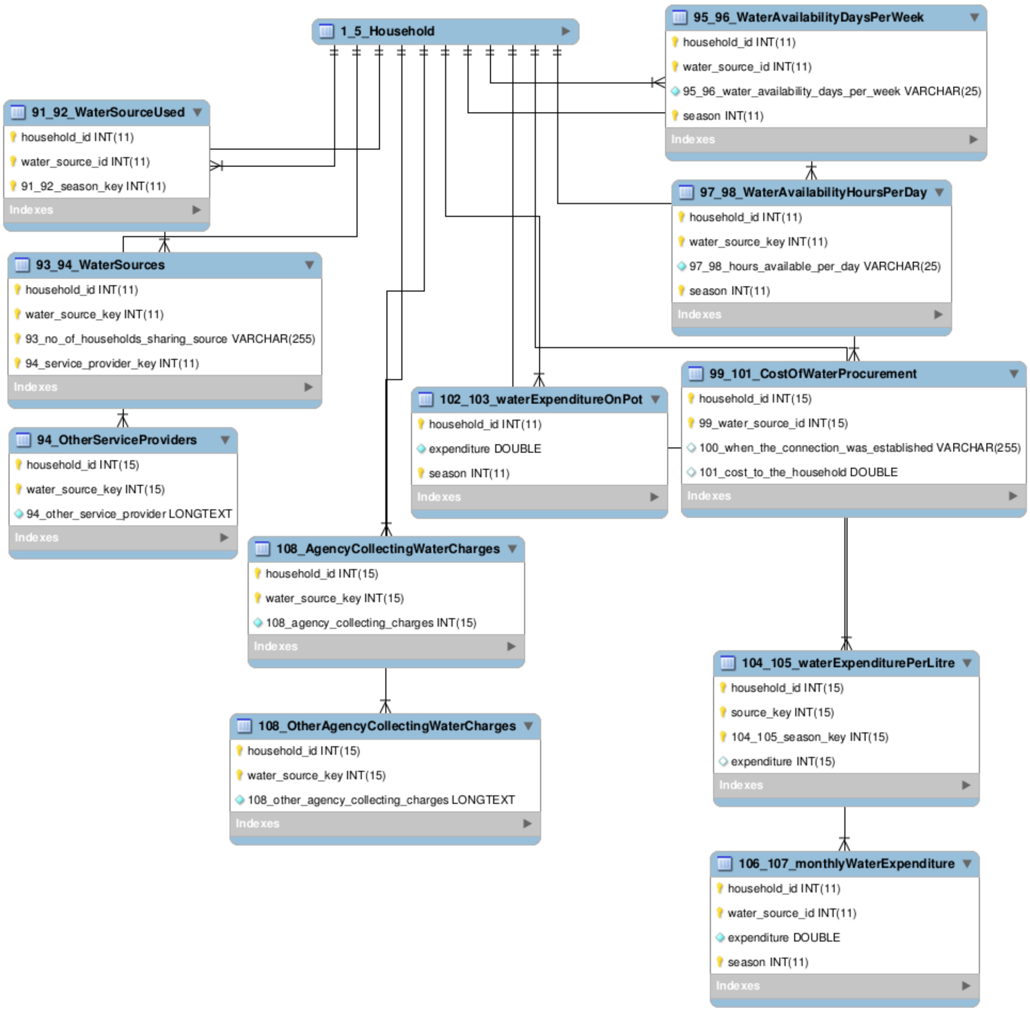

Figure 3.5: Water (Questions 91 to 108)

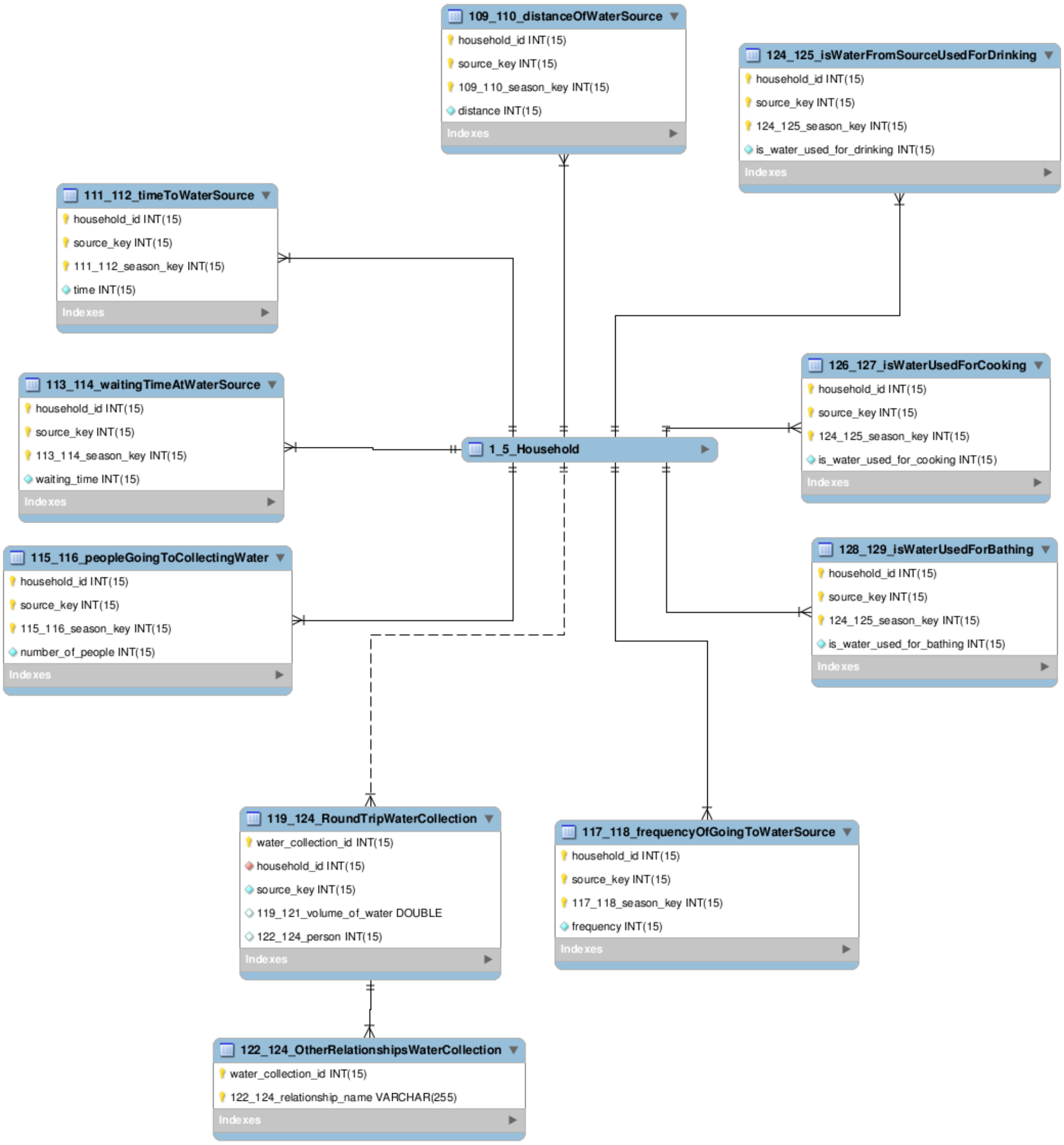

Figure 3.6: Water (Questions 109 to 129)

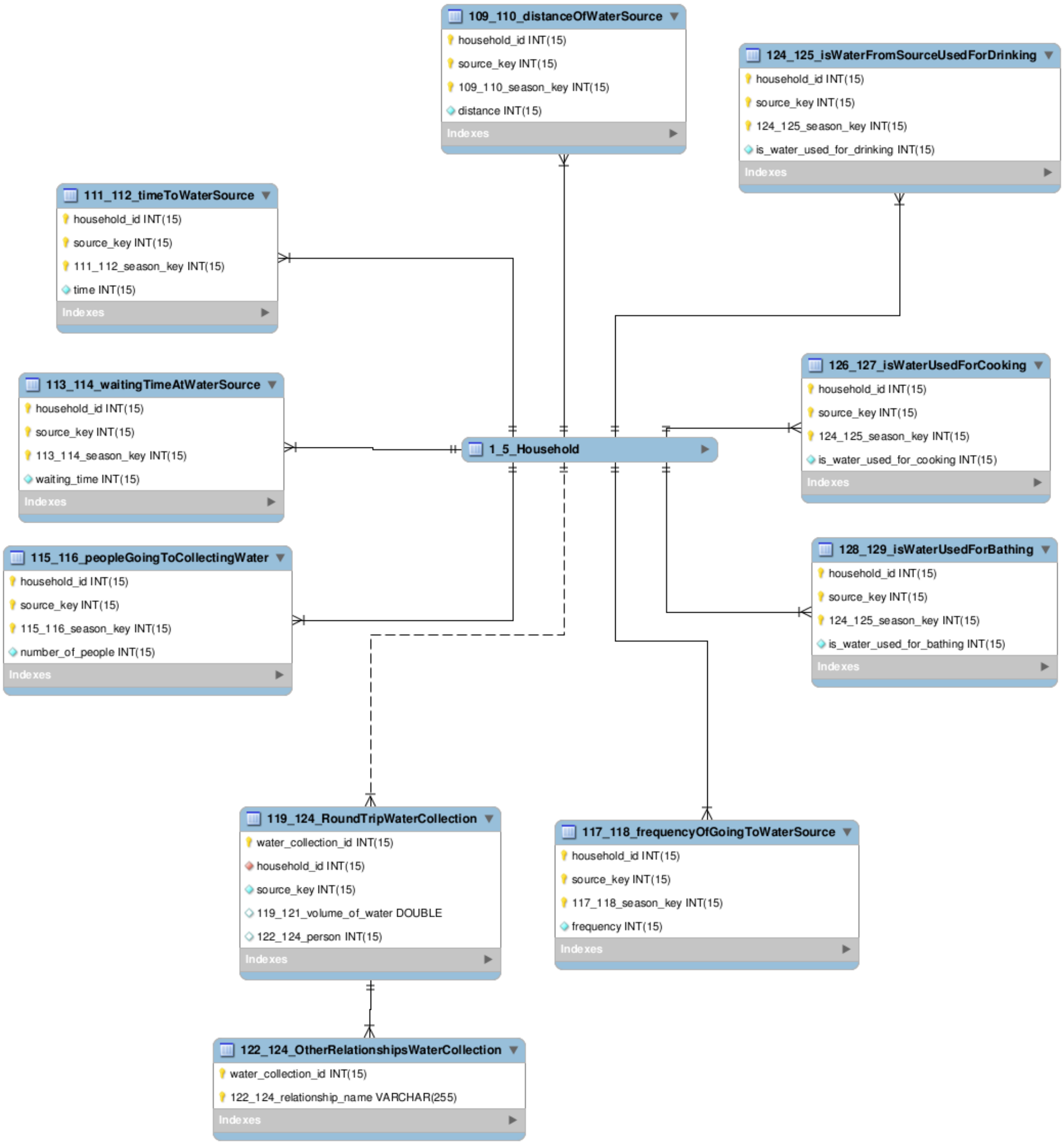

Figure 3.7: Water (Questions 130 to 143)

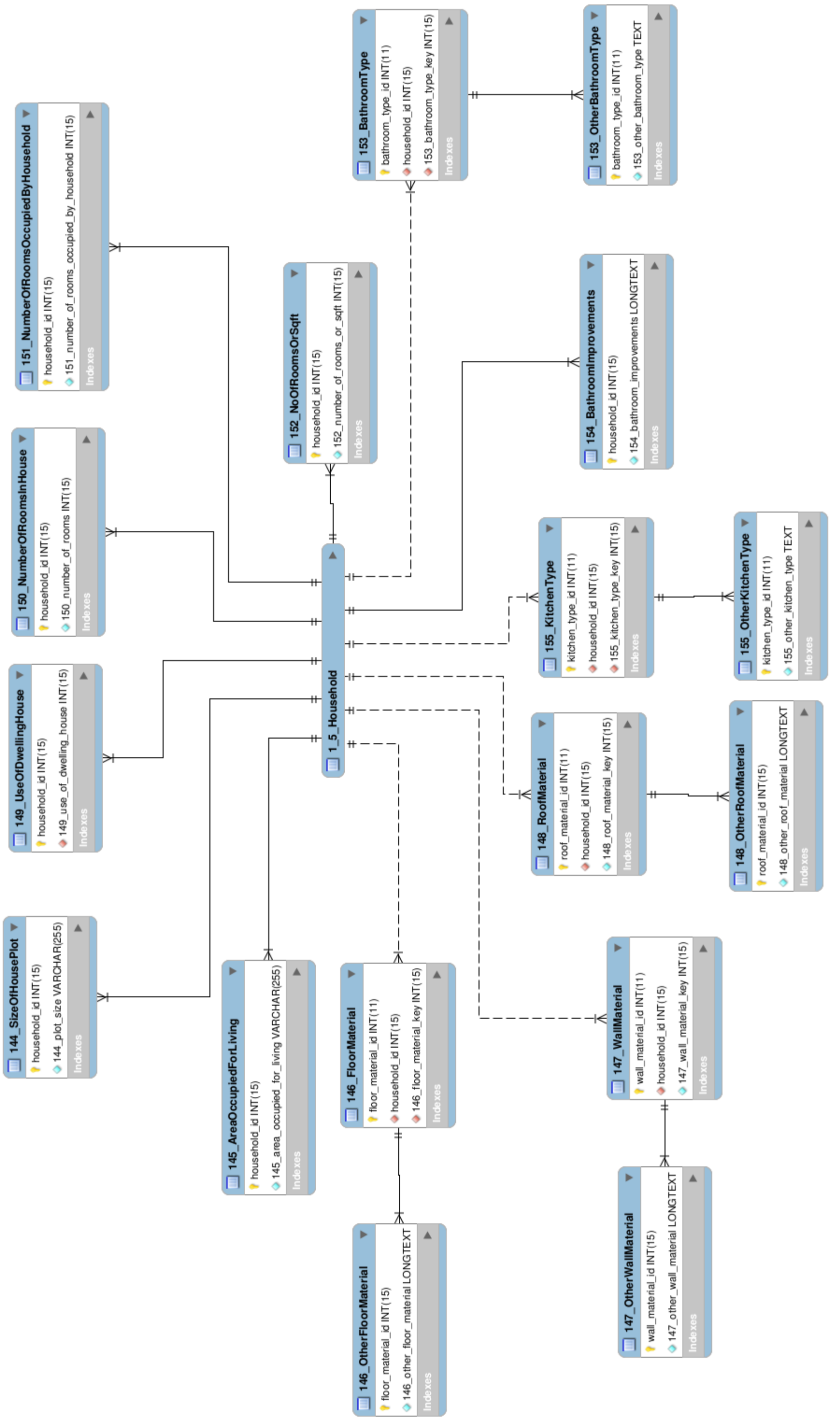

Figure 3.8: Housing (Questions 144 to 155)

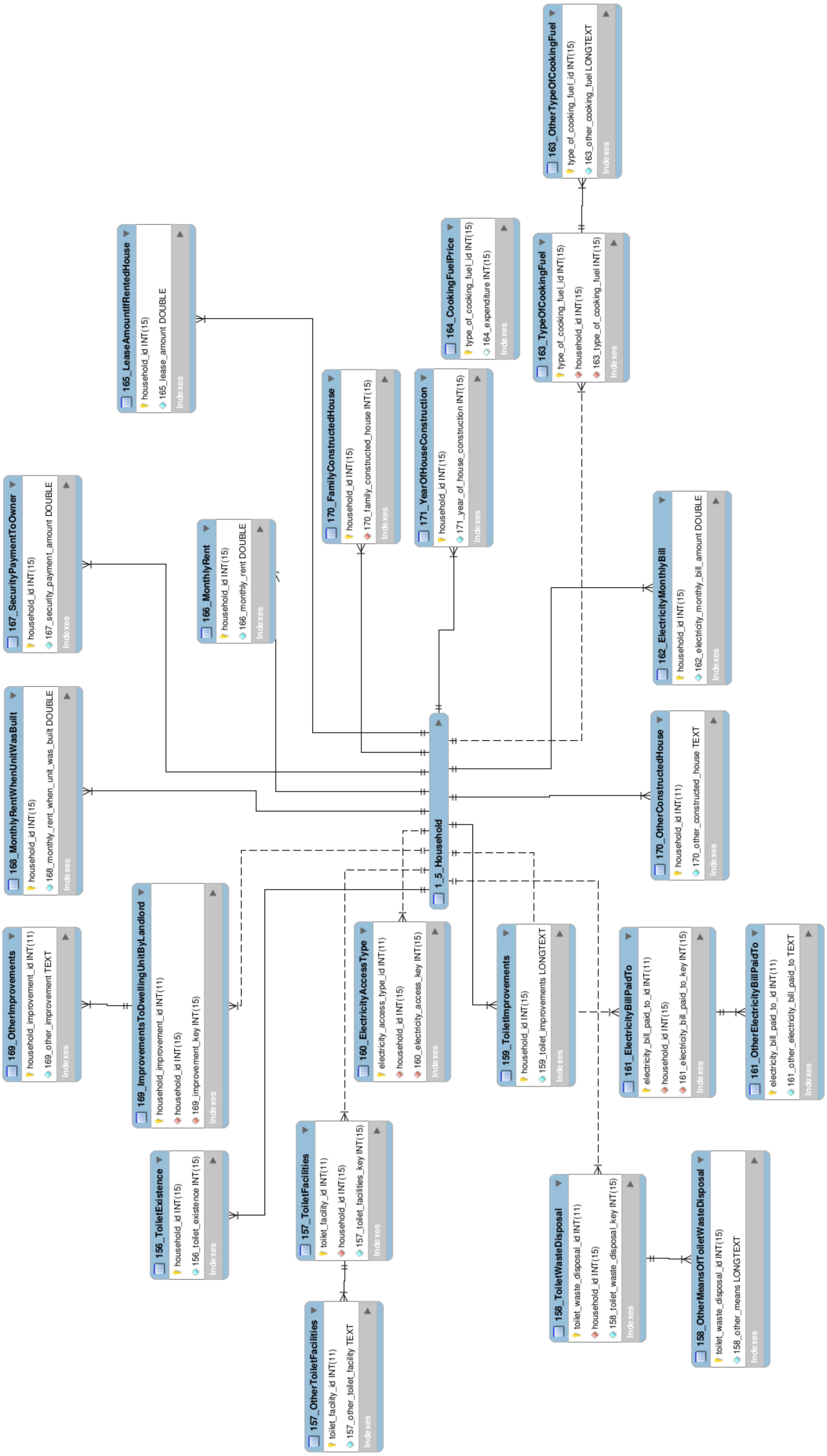

Figure 3.9: Housing (Questions 156 to 171)

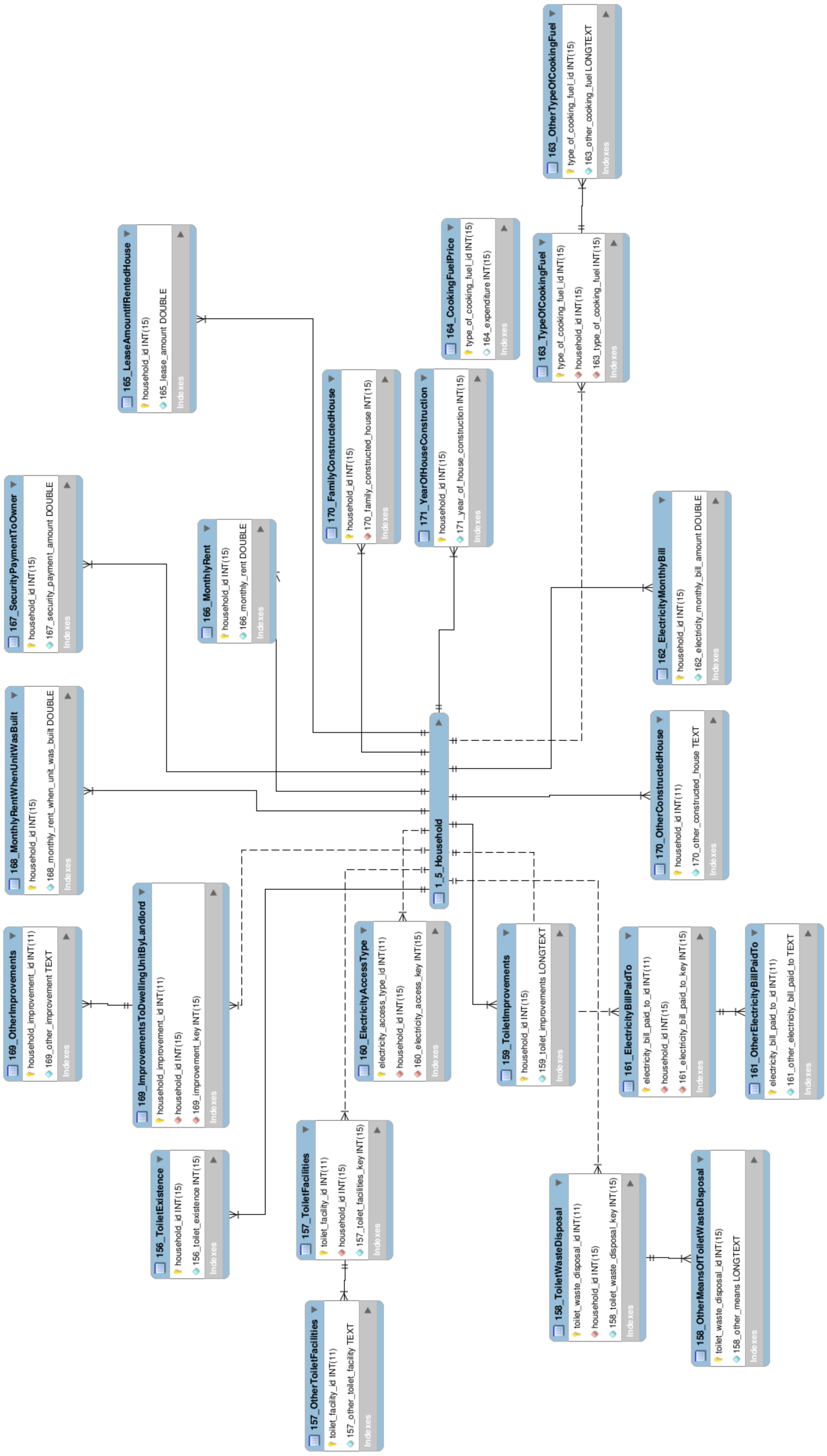

Figure 3.10: Housing (Questions 172 to 189)

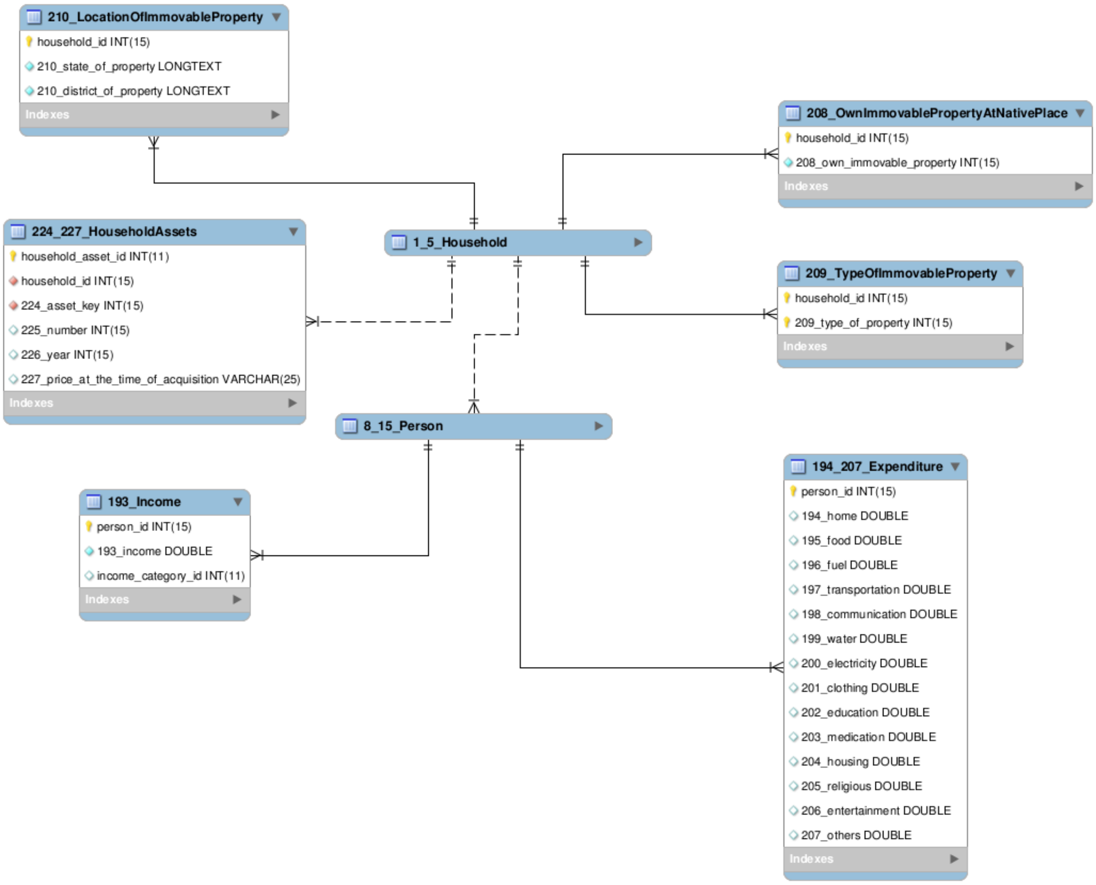

Figure 3.11: Income, Expenditure and Assets

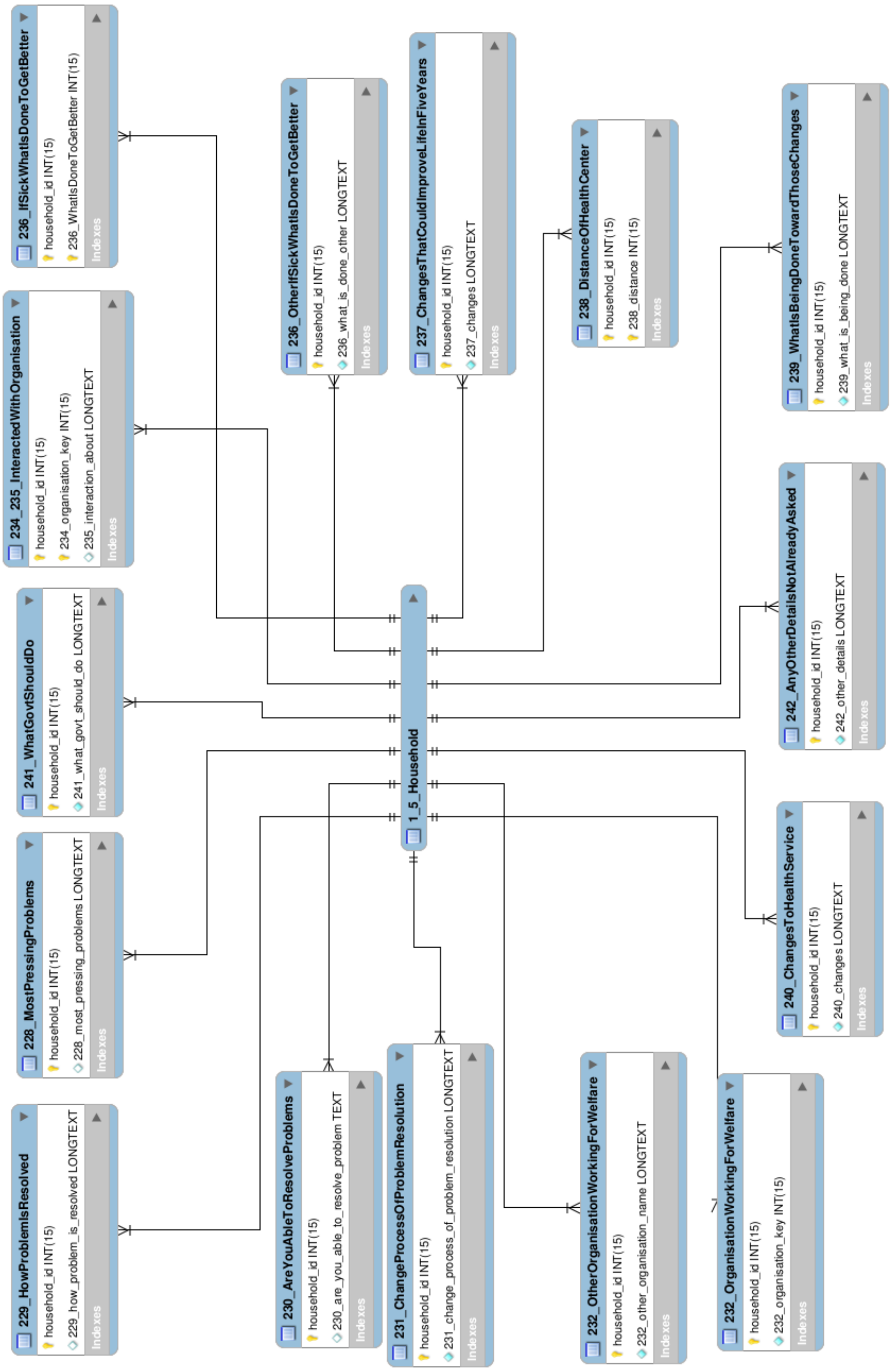

Figure 3.12: Issues, Agencies and Benefits

4 | Construction of variables

Along with the existing variables in the data set, more variables were constructed to help in the analysis. This chapter describes in detail the processes followed in constructing these variables, the results from the different iterations, and the final definition of these constructed variables.

The following sections describe these variables which were constructed using the collected data. Section 4.1 describes the process of categorisation of the households based on their composition and on their earning potential. Section 4.2 describes the process of categorisation of the different occupations reported by the respondents into different occupation categories. Section 4.3 provides the definition of Kutcha and Pucca housing, and the reasons for their differences with the National Sample Survey Organisation definitions. Section 4.4 describes how the income data (individual and household income) was categorised into different bins and Section 4.6 describes the process for defining the age groups. Section 4.7 describes the process that was followed to ascertain the age of the slums.

4.1 Family Categories

Familes are categorised according to their composition and by their earning potential.

4.1.1 First version of the categorisation

The following was the first version of the family categorisation based on composition:

1. **Married Couple with Children:** Households which consist of a couple and their children fall under this category. If these households had multiple heads of household, then they fall under category 3.
2. **Married Couple without Children:** Households which consist of a married couple without any children fall under this category.
3. **Joint Family with Children:** Households with multiple heads of household fall under this category.
4. **Joint Family without Children:** Households with multiple heads of household, but with no children, fall under this category.

5. **Female Householder with Children:** Households with head of household being female, or when the male head of household does not stay in the house, fall under this category.
6. **Householder Living Alone:** All single member households fall under this category.

The following was the first version of the family categorisation by their earning potential:

1. **Female** headed **nuclear** family with **one earning** member
2. **Female** headed **nuclear** family with **multiple earning** members
3. **Female** headed **joint** family with **one earning** member
4. **Female** headed **joint** family with **multiple earning** members
5. **Other-headed nuclear** family with **one earning** member
6. **Other-headed nuclear** family with **multiple earning** members
7. **Other-headed joint** family with **one earning** member
8. **Other-headed joint** family with **multiple earning** members
9. Single households

4.1.2 Second version of the categorisation

The following concerns arose from the first version of the categorisation:

- The above categorisation does not provide a definition for Joint and Nuclear families

- The categorisation by composition and categorisation by earning potential do not follow the same categorisation criteria. The categorisation by composition was done with the primary criteria being whether the families were nuclear or joint, whereas the categorisation by earning potential was done with the primary criteria being whether the household was male or female headed.

Based on these, a second version of the categorisation was done. The following are the definitions being used:

Nuclear Family A nuclear family consists of a married couple and their dependent children. A nuclear family can have multiple earning members only if both the married members in the family earn. The household is a joint family if the children earn as well.

Joint Family A joint family is one where the household consists of more than one adult (not married to each other) and/ or multiple earning members.

Female headed household Households which have reported a female member as the head of the household, or households which have a female member as the primary earning member, are classfied as Female Headed households.

Male headed household Households which have reported a male member as the head of the household, or households which have a male member as the primary earning member, are classfied as Male Headed households.

The final categorisation is based on female-headness as the first criteria, nuclear/ joint family as the second criteria and the number of earning members as the third criteria.

The final categorisation based on family composition is:

1. **Female headed nuclear family**
2. **Female headed joint family**
3. **Female, Single**
4. **Male headed nuclear family**
5. **Male headed joint family**
6. **Male, Single**

The final categorisation based on family composition is:

1. **Female** headed **nuclear** family, **single earning** member
2. **Female** headed **nuclear** family, **multiple earning** members
3. **Female** headed **joint** family, **single earning** member
4. **Female** headed **joint** family, **multiple earning** members
5. **Female, Single**
6. **Male** headed **nuclear** family, **single earning** member
7. **Male** headed **nuclear** family, **multiple earning** members
8. **Male** headed **joint** family, **single earning** member
9. **Male** headed **joint** family, **multiple earning** members
10. **Male, Single**

Households in which the members had not reported any income would be categorised only be their composition (female-headedness) and not by their earning potential. Also, households which have not reported a head of household *would not fall under any of these categories*.

4.2 Occupation Categories

During the survey, the respondents were asked their occupations, and this was recorded without coding. These occupations were then categorised during data clean-up. The categorisation underwent 7 iterations.

First version The first version of the categorisation is given in Table 4.1. It was based on the categorisation by Mitra & Tsujita (Mitra & Tsujita, 2006) in their work on slums in Delhi. This study had defined the following categories:

1. Semi-Professional
2. Sales
3. Trade

4. Personal Services
5. Manufacturing
6. Repairing
7. Commercial
8. Security
9. Transport
10. Tailoring
11. Construction

Table 4.1: Occupation Categories - version 1

Sl.no	Category name	Occupations
1	**Semi professional**	Clerk, Computer Operator, Engaged In Field Work, Government Service As Typist, Owner Of A Health Clinic, Supervisor In A Company, Supervisor in Ngos, Teaching And Giving Tuition, Technical Assistant In Air India, ICICI Bank Agent, , Data Entry, Courier, Cashier, Airtel Executive, Accountant, Account Assistant.
2	**Sales**	Selling Books, Magazines And News Papers, Egg Seller, Working In A Garment-Exporting Agent, Washing Clothes In A Garment Exporting Agent, Stock Checking, Fish Vender, Flower Vender, Fruit Packing In Wholesale Market (Mandi), Working In A General Store, Helper In A Store, Helper In An Export Agent, Helper In A Foot-Wear Shop, Helper In A Chemical Store, Helper In A Shop, Helper In An Export Company's Shop, Helper In A Garment-Export Shop, Helper In A Fruit Mandi, Helper In A Garment Shop, Helper In A General Store, Helper In A Hardware Shop, Helper In Indian Airlines, Helper In A Shop Selling Jeep Battery, Helper In A Juice Shop, Helper In A Medicine Shop, Helper In A Company Selling Snacks (Namkeen), Helper In A Shop Selling Sauce, Helper In A Shop Selling Tv's, Helper In A Shop Selling Wood Work, Ice Cream Vender, Collecting Garbage And Waste (Kabariwala), Peanut Seller, Pan Seller, Seller Of 'bidi' And Cigarettes On The Road, Salesman (Medicine, Cold-Drinks, Etc), Selling Vegetables, Selling Wood, Dealing With Sale And Purchase Of Cars, Sweet Vender, Working In The godown Of Waste And Garbage Collection, Serial Set, Shamiyana Work, All Sales, Shops, Business, Butcher, Computer Sales, Cd Casette Work, Petrol Bunk Helper, Scrap Work.

Occupation Categories - version 1

Sl.no	Category name	Occupations
3	**Trade**	Trading In Cloth, Fisherman And Trading In Fish, Trading In Hosiery, Helper In An Iron/Steel Shop, Helper In A Sweet Shop, Helper In A Workshop, Working In A Hotel, Providing Room Service In Hotels, Working In Shops, Suitcase Fitting, Working In A Tea Shop, Owning A Tea-Stall, Working In A Hotel, Working In Readymade Garment Shop, Working In A Canteen. Making Coconut Barfi, Bakery, Beauty Parlor, Cleaning Vessels In Hotel, Cloth Sales, Making Tiffin At Home, Waiter, Saree Sales.
4	**Personal Services**	Barber, Basti Sewika (Paid Social Worker), Cleaning Utensils And Washing, Cook, Traditional Mid-Wife, Domestic Maid Or Servant, Helper In A Kitchen, Gardener In A Farm House, Serving Drinking Water In mandi, Sweeper And Working In Small Eating Places (Dhaba) Or Tea Stalls As A Cleaner Or Sweeper, Ayamma, Anganwadi Teacher, Chowltry Worker, Sweeper
5	**Manufacturing**	Bamboo Work, Box Making, Bricks Making Unit, Bulb Factory, Candle Making, Manufacturing Decorative Items Made Of Paper, Factory Worker, Foreman, Furniture Work, Glass Work, Helper In A Plastic Factory, Helper In A Factory, Helper In A Mineral Water Factory, Helper In An Iron Factory, Helper In A Plastic Factory, Helper With A Printing Press, Helper In A Rubber Factory, Helper In A Leather Factory, Labor In An Iron Factory, Lamination Work, Operator, Printing Job, Printing Press, Working In Readymade Cloth Manufacturing Units, Screen Printing, Steel Almira Work, Supervisor In A Steel Factory, Tube Light And Bulb Factory, Utensil Polish Work, Wood Work, Woolen Work, Working In A 'Bidi Company', Working In A Foot-Wear Factory, Working In An Electric Shop, Manufacturing Of Food Products And Working In A Radio And Tv Parts Company, Photo Frame Work, Reshme Work, Toy Making, Iron Bar Work , Stitching Carry Bags, Cover Washing, Coolie, Tyre Coolie, Company<br>Coolie, Book Binding , Beedi Making, Agarbathi Making, Agarbathi Packing, Attender, Chappla Making, Chimta Making, , Carrying Sacks.

Occupation Categories - version 1

Sl.no	Category name	Occupations
6	**Commercial**	Bill Collection, Cable Tv Operator, Courier Service, Helper In An Embassy, Loading Goods, Municipal Corporation Of Delhi (Mcd) Worker, Packaging, Peon In Commercial Units, Class Four Employees/Peon (At Airport, Private Hospital And Mcd Worker), Working In A Video Library And Working In Delhi Electricity Supply Undertaking (Desu), Wall Man, Rmc Carrying, Corporation, Paurakarmika, Toilet Cleaner, Carrying Luggage
7	**Transport**	Auto-Rickshaw And Tempo<br>Driver, Conductor, Driver (Car, Bus, Truck Etc.), Helper In The Transport Sector, Helper In Transporting Goods, Rickshaw Puller And Truck Supervision. Carrying Luggage
8	**Tailoring**	Embroidery, Stitching And Tailoring (Tailor Master And Worker Both), Coloring Thread And Cutting Thread, Saree Work
9	**Construction**	Construction Workers (Beldar, Dehari)<br>Carpenter (Daily Wage Carpenter), Labor In Construction Work, Mason (Mistri), Polishing, Supervisor In Building Construction And Whitewashing, Barbender, Cement Work , Painter, Painting Coolie, Plumber, Rod Bending, Store Work, Tiles, Roofing Construction
10	**Security**	Security Guard, Watchman
11	**Repairing**	Car Mechanic, Cycle Repairing, Electrician, Fitter, Auto Mechanic, Learning Electric Work, Machine Repairing Work, Mechanic, Mechanic Of Electronic Items, Motor Fitter, And Other Repairing Work, Bucket And Stove Repair, Car Painter, Cycle Shop, Mobile Repair

Some occupations, however, could not be categorised. For instance, snake charmers did not fall under any of the categories.

Second version It was found that even after incorporating occupations from this study into the existing categorisation, it could not account for a large range of occupations in domestic services and home based manufacturing. Thus, these two new categories were created.

Third version Since Trade and Self-employment were in the same category, the occupations which were reported as self employed (during the survey) were identified for further analysis in the next iterations.

Fourth version Sweepers, paura karmikas, corporation work, and toilet cleaners were moved to manual labour category, from domestic services. Coolies, painters and plumbers now fell under manual labour so as to differentiate between manual labour and construction work.

Fifth version Mesthri was moved to semi-professional category from construction work as this was a supervisory role in construction work. Pigmy was moved from self-employed category to semi-profesional. "Mysore sandal factory supervisor" was moved to professions. Agarbathi packing was moved to home based manufacturing. Tailor, mobile repair and motor repair were moved to self-employed category. Plumber and painter were moved to construction work from manual labour.

Sixth version A separate category called Sales and Small Enterprise was created, with occupations such as tea powder making, book binding and bakery work. Domestic Services was renamed to Domestic and Cleaning Services to include housemaids (who work at homes) and house keeping (who work as staff at various establishments such as colleges, malls etc.). Manufacturing and repair work were split into two categories as well, as repair work falls under services.

Final version The first category, "Professional and Semi professional" was split into Professional White Collar and Professional Blue Collar as the income differences between the two were significant. The final categories are given in Table 4.2.

Table 4.2: Occupation Categories - Final version

Sl.no	Category name	Occupations
1	**Professional White Collar**	Accountant, Account Assistant, Airtel Executive, Anganwadi Teacher, Attender, Architect, Bank money pigny, Bank and related, call center, cashier, computer graphics, computer sales, computer office, computer typist, courier counter staff, data entry operator, Engineer, Field work, Estate finance, Finance office, Gym coacher, Hard ware engineer, Company assistant, Mysore sandal factory supervisor, Factory supervisor, NGO, Nurse, Office Assistant, office clerk, Reliance office, Sales officer, social service, Superior work, Social worker, Sports trainer, Supervisor, Teacher, Telecom office, Traffic police
2	**Professional Blue Collar**	Office, Office boy, private company, Private office, Medical Helper, work in Company, Medical shop, Medical Office, Courier, collection counter, Water man, Wall man BBMP, Water meter reader, Attender

Occupation Categories - Final version

Sl.no	Category name	Occupations
3	**Sales and Small enterprises**	Tea powder making, Bakery work, Book binding, Cable worker, Chicken work, Cloth sales, Cook in travels vehicle, Cover washing, All sales, Hotel, Hotel master, Hotel supplier, Hotel work, House deliver, Packing, Petrol bunk, Photoframe work, Press work, Ration packing, Screen printing, Store work, Vehicle show room, Waiter, Supplier,, Sales boy, Printing press, Shoe sales, Ticket distributor in theater, Work in gym, Work in hospital, Work in tea shop, Work in vegetable shop, chicken eggs, Tailor in shop, Working in work shop, Working in Mechanic shop, Working in Garage, Working as Butcher, Working as Fish seller, Working as vegetables & Fruit vendors for somebody
4	**Self-employed**	Bed & sofa making, Actor, Aluminium shop, Bakery, Barber, Beauty parlor, Business, Butcher, Canteen, CD casette work, CD work, chicken shop, cloth shop, coconut selling, fish sales, flower sales, fishmonger, Foot wear shop, Fruit seller, Furniture, Hardware shop, Idli shop, kerosene depoy, Making Statues, Symbol making, Mobile canteen, Mobile shop, Mutton shop, Plastic sales, Petty shop, Plastic business, Pooja material sales, Push cart, Reshme work, Rudraksha business, Rudraksha sales, Scrap shop, Scrap business, Snack business, Snake charmer, Spare parts business , selling toys, Silver vessel shop, Soap business, Sponge sales, steel scrubber sales, Tamte, Tender coconut business, under garment business, utensils business, Vegetable seller, Vegetable vendor, Tea business, Tailor shop, Tea shop, Tool shop, All vendors, Waste material sales, Water seller, Sim card sales, Scrap work
5	**Self-Employed Repair and Service**	Plastic repair, Mobile repair, Motor repair, Bucket & stove repair, News paper distributor, Electronic work, Tinker, Work shop, Motor work , Cable operator, cycle shop owner, cobbler, Horse shoe fixing, gold work, Ironing, Lock repair, Mechanic shop, Piano business, Serial set, Shamiyana work, Tailor, Chappal making, Car mechanic, Car painter, Auto garage, Auto mechanic, Lift mechanic, Lorry mechanic, Mechanic, garage, Vehicle painting
6	**Home-based employment**	Agarbathi Making, Beedi making, Doing chimtas & Ear cleaning sticks, Embroidery , Making bags, Making coconut barfi, Making tiffin at home, Saree work, Sandige papad business, Tailor in house, Toy making

Occupation Categories - Final version

Sl.no	Category name	Occupations
7	**Domestic and cleaning services**	Aya, All types of cooks, Dish wash, Gardener, Garden coolie, Hospital work , House maid, House keeping, School maid, Washerman, Sweeper in corporation, sweeper in shops, Toilet cleaner, Lorry cleaner, Chowltry work
8	**Manufacturing and Small Industry**	Furniture work, Garments helper, Agarbathi factory, Agarbathi Packing, Almirah making, Aluminium, Automobile, Bearing, Cargo box operator, Company, factory, Greasing : Lorry, HMT, HAL, Leath work, Electronic work, Garment , Tailor in garment, Net factory, Oil factory, Service station, Shoe factory, Steel factory, Wood work , Wood polish
9	**Security**	Security, Security officer, Watch man
10	**Transport services**	Auto driver, Car driver, Driver, Luggage vehicle driver, School van driver, Tempo driver
11	**Construction**	Barbending, Carpenter, Centring, Cement work, Construction all types, Iron bar work, Filling sand, Roofing, Tiles, Mesthri, Mico, Electric and Electrician, Welding, and Plumber, Painter, Joining people for Painting work, Mason, Concrete, Cement mixing, Construction supervisor
12	**Manual Labour**	Bharath gas, Carrying sacks, Cleaning bottles, Cleaning vessels in hotel, Loading & unloading, Helper, Carrying luggage, RMC , Gunny bag stitching, Pete coolie, All types of coolie, Digging pit in cemetry, Picking paper, Corporation work, Paurakarmika, Vehicle coolie, Contract labor, Coolie, Loading mud, Labor, Mud exacavation, Chapadi stone work, Company coolie, Scrap shops (carrying sacks), Filling mud, Filling sack, Carpenter Coolie, Cover washing, Coolie in Hotel, Coolie, Coolie in shop, Sheet Arrangement, Tyre Coolie, Coolie in Sales

4.3 Housing

The National Sample Survey Organisation (NSSO, 2001)defines the following housing structures:

1. Pucca structure: A pucca structure is one whose walls and roofs are made of pucca materials such as cement, concrete, oven burnt bricks, hollow cement/ ash bricks, stone, stone blocks, jack boards (cement plastered reeds), iron, zinc or other metal

sheets, timber, tiles, slate, corrugated iron, asbestos cement sheet, veneer, plywood, artificial wood of synthetic material and poly vinyl chloride (PVC) material.

2. Kutcha structure: A structure which has walls and roof made of non-pucca materials is regarded as a katcha structure. Non-pucca materials include unburnt bricks, bamboo, mud, grass, leaves, reeds, thatch, etc. Katcha structures can be of the following two types:

   (a) Unserviceable katcha structure includes all structures with thatch walls and thatch roof i.e. walls made of grass, leaves, reeds, etc. and roof of a similar material and

   (b) Serviceable katcha structure includes all katcha structures other than unserviceable katcha structures.

3. Semi-pucca structure: A structure which cannot be classified as a pucca or a katcha structure as per definition is a semi-pucca structure. Such a structure will have either the walls or the roof but not both, made of pucca materials.âĂİ

However, some households in this survey have both, the walls and the roofs as asbestos sheets. As asbestos walls are not stable and are likey to fall without adequate support, a house ought to be categorised as pucca only if the roof is made of asbestos, but not the walls. Such type of housing cannot be classifed as pucca housing, according to the NSSO classfication.

To address such issues, the definitions of kutcha, pucca and semi-pucca housing was modified as:

1. Pucca structure: A pucca structure is one whose roofs are tiles and walls are wood, G.I/ metal/ asbestos sheets *or* whose roofs are stone, concrete, and wall is wood, G.I/ metal/ asbestos sheets, burnt brick, stone, concrete, cement bricks.

2. Semi-pucca structure: A semi pucca structure is one whose roofs are tiles, stone, concrete and wall is grass/ thatch/ bamboo, plastic/ polythene, mud/ unburnt brick, wood, G.I/ metal/ asbestos sheets *or* whose roofs are grass/ thatch/ bamboo, plastic/ polythene and walls are burnt brick, stone, concrete, cement bricks.

3. Kutcha structure: A kutcha structure is one whose roofs are grass/ thatch/ bamboo, plastic/ polythene, G.I/ metal/ asbestos sheets and walls are grass/ thatch/ bamboo, plastic/ polythene, mud/ unburnt brick, wood, G.I/ metal/ asbestos sheets.

4.4 Income Categorisation - Individual Income

Out of the data of the 5634 people in the survey, 2049 people reported their income. As seen in Fig 4.1, the income distribution resembled a long-tail distribution. The income categorisation had to be chosen which best represented this distribution.

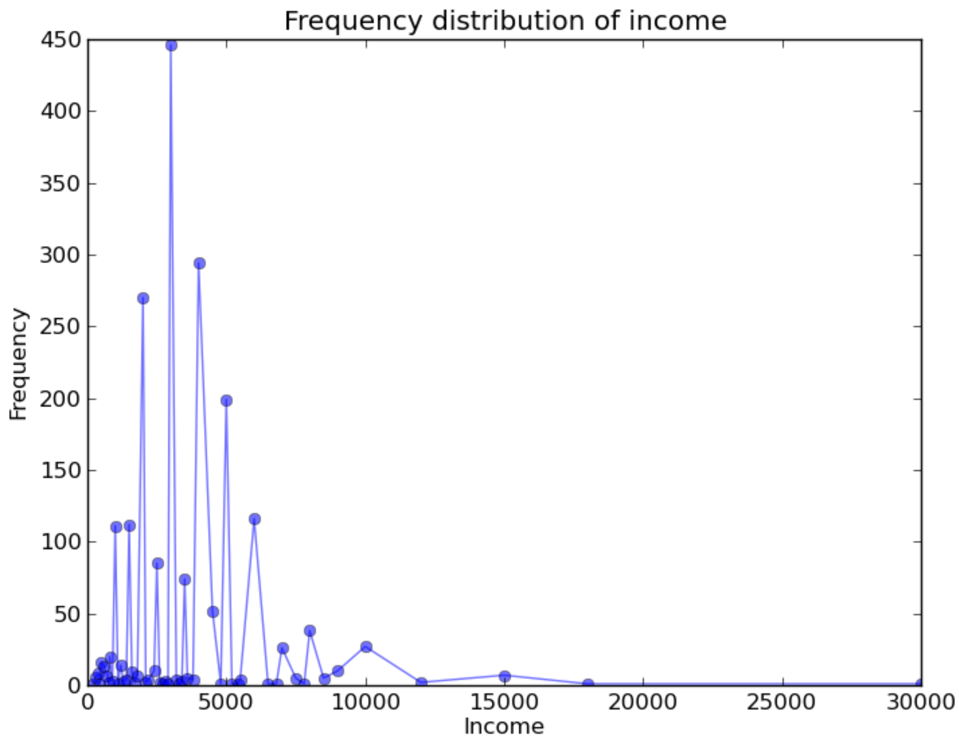

Figure 4.1: Income Distribution in the data

4.4.1 First version of the categorisation

To identify the intervals and categories of the income, the following process was followed:

1. Clustering to identify possible interval sets: *k-means clustering* was used on the income vectors to identify clusters and intervals. This clustering algorithm was run three times to identify 3, 4, 5, 6, 7, 8, 9 and 10 clusters, and their respective intervals. This was performed to identify the number of intervals we would require to represent the income distribution.

2. Identifying interval sets which best represent a long-tail distribution: From the results of the clustering, the following interval sets were identified as best representing a long-tail distribution:

   (a) [200,1600), [1600,2400), [2400,3600), [3600,4800), [4800,6000), [6000,8000), [8000,30000]: The frequency chart is shown in Fig 4.2.

   (b) [200,1300), [1300,2400), [2400,3600), [3600,4800), [4800,6000), [6000,8000), [8000,30000]: The frequency chart is shown in Fig 4.3.

   (c) [200,1300), [1300,2400), [2400,3500), [3500,4600), [4600,5700), [5700,8000), [8000,30000]: The frequency chart is shown in Fig 4.4.

3. Narrowing down from the selection to one interval set: The interval set number 3 (Fig 4.4) was chosen as it best represented a long-tail distribution.

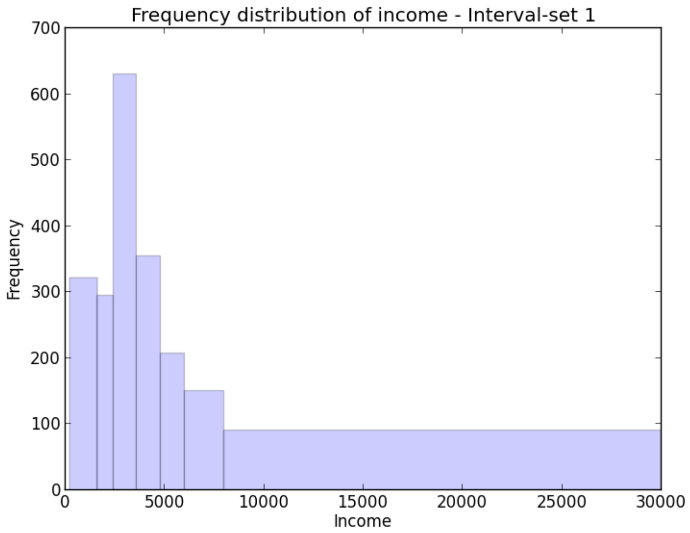

Figure 4.2: Frequency Distribution for Income Intervel-Set 1

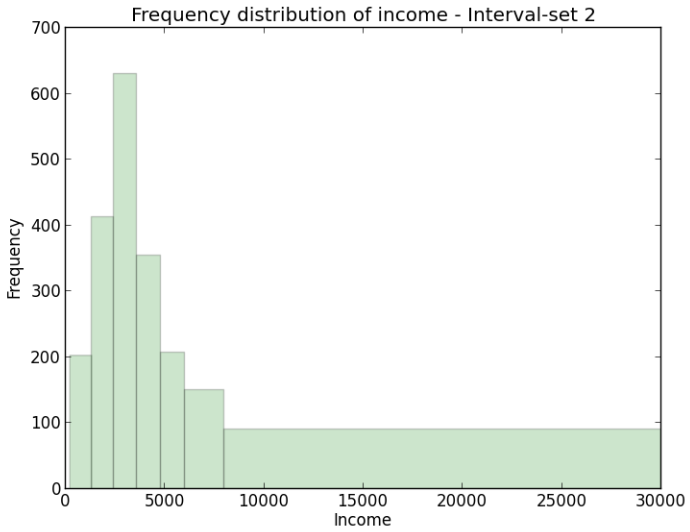

Figure 4.3: Frequency Distribution for Income Intervel-Set 2

4.4.2 Second version of the categorisation

The interval [8000-30000) was split into [8000-12000), [12000-18000) and >18000, as the interval range was too large.

The final income intervals chosen were:
[200,1300), [1300,2400, [2400,3500), [3500,4600), [4600,5700), [5700,8000), [8000,12000),[12000,18000), >18000

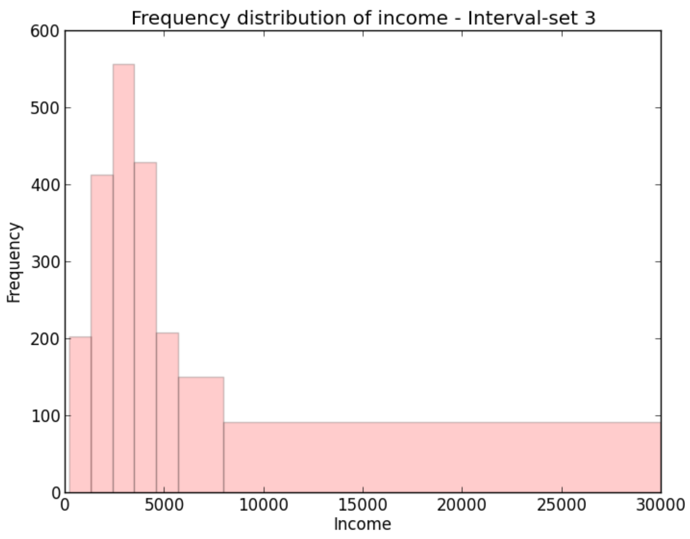

Figure 4.4: Frequency Distribution for Income Intervel-Set 3

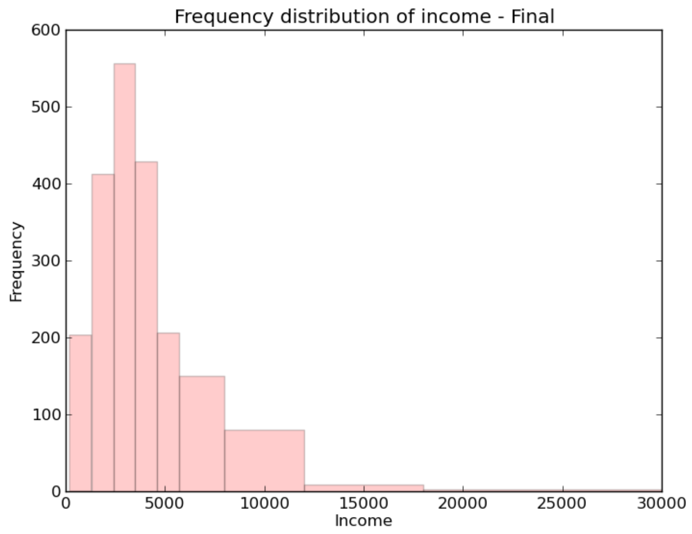

Figure 4.5: Frequency Distribution for Income Intervel - Final

4.5 Income Categorisation - Household Income

Household income is calculated by calculating the sum of the individual incomes in the household. Similar to individual incomes, the household income distribution resembled a long-tail distribution (Fig 4.6). The income categorisation had to be chosen which best represented this distribution.

1. Clustering to identify possible interval sets: *k-means clustering* was used on the income vectors to identify clusters and intervals. This clustering algorithm was run three times to identify 3, 4, 5, 6, 7, 8, 9 and 10 clusters, and their respective intervals. This was performed to identify the number of intervals we would require to represent the income distribution.

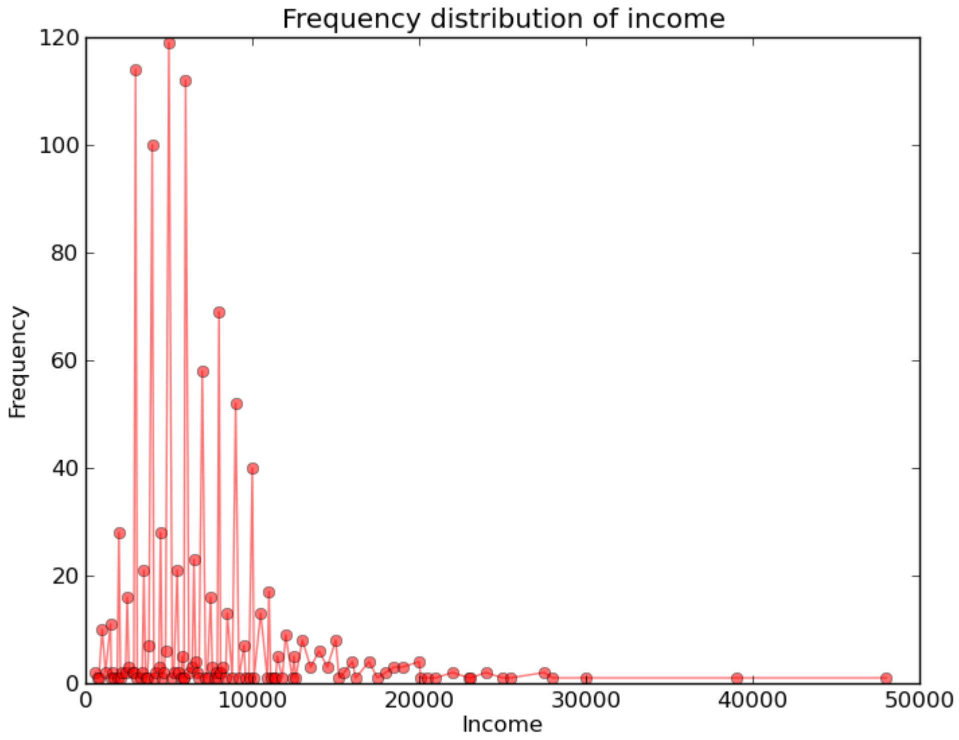

Figure 4.6: Household Income Distribution in the data

2. Identifying interval sets which best represent a long-tail distribution: From the results of the clustering, the following interval sets were identified as best representing a long-tail distribution:

   (a) [600,2600), [2600,4500), [4500,6800), [6800,9600), [9600,15500), [15500,48000]: The frequency chart is shown in Fig 4.7.

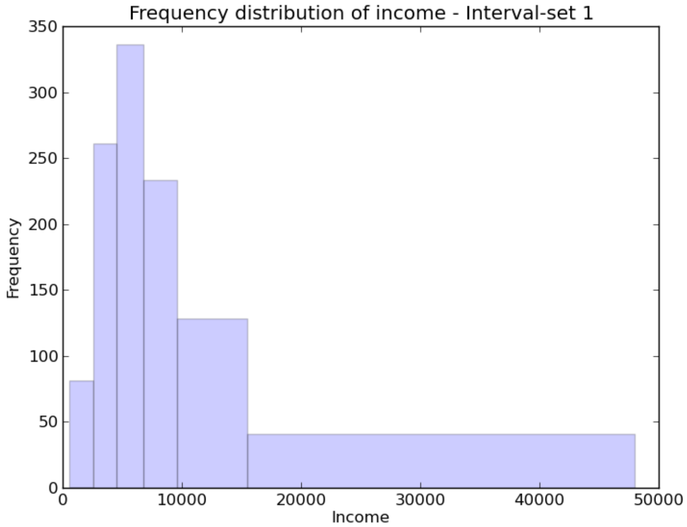

Figure 4.7: Frequency Distribution for Income Intervel-Set 1

(b) [600,2600), [2600,4500), [4500,6800), [6800,10000), [10000,15000), [15000,20000), [20000,25000), [25000,30000), >30000: The frequency chart is shown in Fig 4.8.

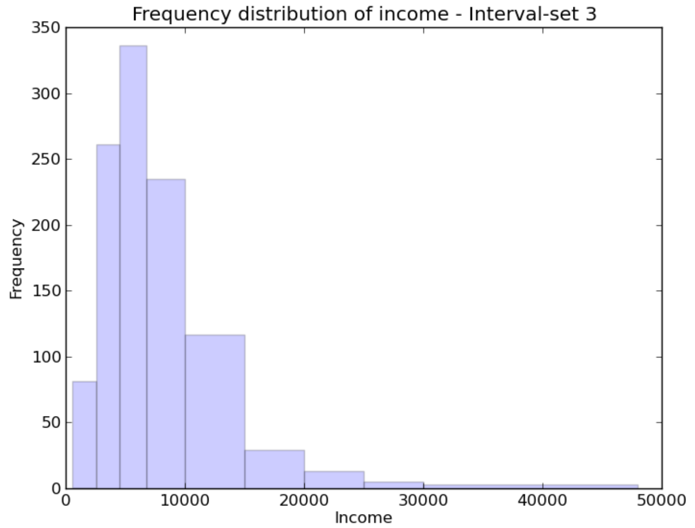

Figure 4.8: Frequency Distribution for Income Intervel-Set 2

4.6 Age Groups

Age categorisation was done based on the requirement that literacy and occupation data need to be analysed according to age. The first categorisation of age data was:
[0, 6), [6, 10), [10, 14), [14, 18), [18, 25), [25, 30), [30, 40), [40, 60), [60, 80), [80, 95)

Figure 4.9 shows the frequency distribution for this interval set.

The age group **[0,6)** was added to identify the outliers in occupation data. This category is excluded from analysis of occupation data.

Age groups from 6 to 18 were divided into three bins, **[6,10)**, **[10,14)**, **[14,18)**. This was done to help identify children in these ages groups, their literacy and their occupation (if any) [1].

For the second version of the categorisation, the age group **[18,25)** and **[25,30)** were merged to form **[18,30)**. The age group following this are in increments of 10.

Furthermore, it was identified that splitting the age groups from 6 to 14 into two groups **[6-10)** and **[10-14)** would not be useful when analysing occupation data, but very important to analyse literacy data. Therefore, people were categorised into two age groups, one for literacy data analysis and one for occupation data analysis.

The final age bins are:

[1]Age of majority in India is 18.

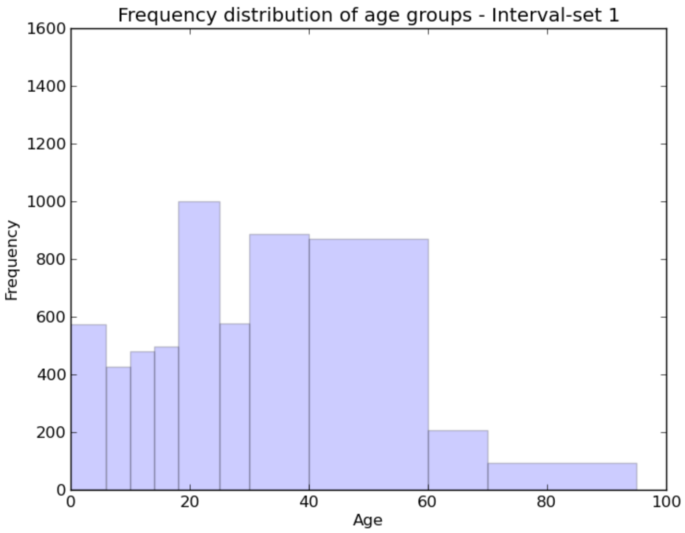

Figure 4.9: Frequency Distribution of age groups, Intervel-Set 1

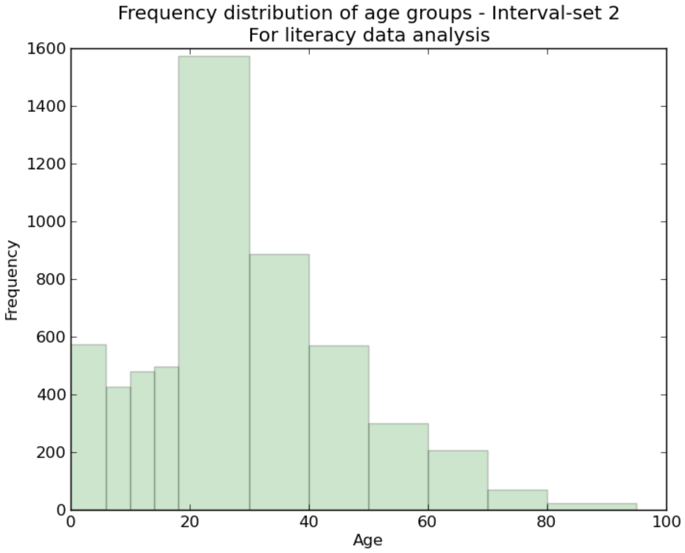

Figure 4.10: Frequency Distribution of age groups, Intervel-Set 2, for literacy data analysis

1. Age Bins for Literacy Data Analysis: **[0,6), [6,10), [10,14), [14,18), [18,30), [30,40), [40,50), [50,60), [60,70), >70**. Figure 4.10 shows the frequency distribution for this interval set.

2. Age Bins for Occupation Data Analysis: **[0,6), [6,14), [14,18), [18,30), [30,40), [40,50), [50,60), [60,70), >70**. Figure 4.11 shows the frequency distribution for this interval set.

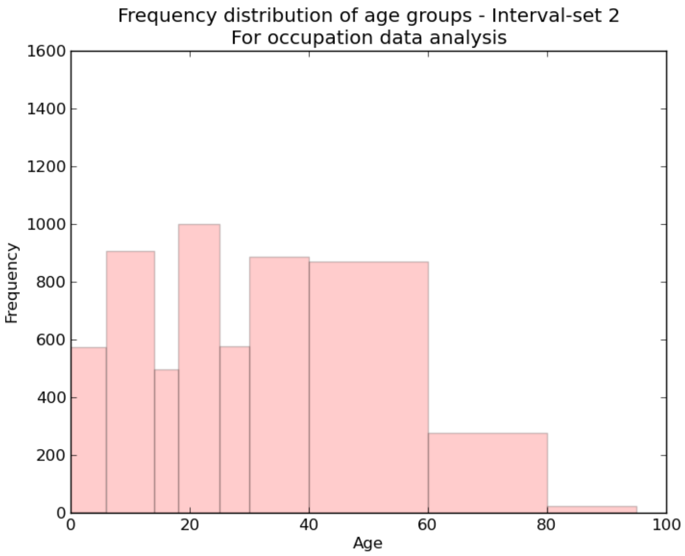

Figure 4.11: Frequency Distribution of age groups, Intervel-Set 2, for occupation data analysis

4.7 Age of the slums

Ages of the slums were required to understand its relationship with the facilities and infrastructure available within these slums, and to understand how slum demographics vary based on whether the slums are rehabilitated, non-rehabilitated, declared, old or new.

The first set of ages of the slums was acquired through a personal interview with Mr. Issac Arul Selva of Slum Jagatthu. The slum ages from the first version of the documentation are given in Table 4.3. This data contains information on when the slum came into existence and when the slums got rehabilitated.

Table 4.3: Age of the slums, version 1

Slum id	Slum name	Age as of 2010
1	ANON	40
2	ANON	Shifted from ANON in 1992.
3	ANON	30 years ago it was a small slum, now 10 years ago it grew to this size
4	ANON	30-35
5	ANON	Before 1990, it was part of ANON slum. After relocation, the slum was named as ANON
6	ANON	70
7	ANON	28
8	ANON	50
9	ANON	25
10	ANON	30

Age of the slums, version 1

Slum id	Slum name	Age/comments
11	ANON	45
12	ANON	Before 2004, it was part of ANON ANON before. After relocation, the slum was named as ANON.
13	ANON	25
14	ANON	25
15	ANON	Before relocation, the slum was near ANON for 25 years. It has been 5 years after relocation.
16	ANON	The slum was 30 years old before getting relocated in 2009.
17	ANON	50
18	ANON	30
19	ANON	Before relocating in the year 2009, the slum was near ANON.
20	ANON	23
21	ANON	The slum is as old as ANON
22	ANON	40
23	ANON	40 years
24	ANON	Before relocation in the year 2006, the slum was 65 years old and was at ANON.
25	ANON	35
26	ANON	20
27	ANON	30
28	ANON	35
29	ANON	60
30	ANON	50
31	ANON	The slum came into existence during the period of Devaraj Urs as Chief Minister of Karnataka.
32	ANON	40
33	ANON	1975 (38)
34	ANON	50
35	ANON	
36	ANON	This is a gypsy/ nomadic slum, which was 13 years old before shifting to ANON.

The case studies carried out during the survey were referred to look for details of the age of the slum. Table 4.4 shows the data obtained through the case studies.

Table 4.4: Age of the slums as identified from case studies

Slum id	Slum name	Age as of 2010
4	ANON	The respondents said that it would be difficult to say when this area came into existence as it was over 30 years since they began staying there.
5	ANON	The respondents said that they have been given houses at Laggere slum as rehabilitation 9 years ago. It has been 20 years or more since they moved to Haavadigara colony.
6	ANON	This has been agricultural land since 1951 and the respondents have been staying here since the Indian Independence
14	ANON	The respondents said that this slum cam into existence in 1995
17	ANON	The respondents informed that the slum has over 100 years of history and that the British soldiers used to go horse riding there
18	ANON	The respondents said that it has been over 20 years since they moved to the slum.
34	ANON	The slum came into existence over 30-35 years ago.
36	ANON	The repondents said that the slum started in 1992

Table 4.5 shows the slum ages according to Karnataka Slum Clearance Board surveys of 1994, 2008 and 2010.

Table 4.5: Age of the slums as per KSCB surveys

Slum id	Slum name	KSCB 1994	KSCB 2008	KSCB 2010
1	ANON			30
2	ANON			40-50
3	ANON			
4	ANON		30 (1980)	
5	ANON			9
6	ANON		50 (1960)	
7	ANON		30 (1980)	21
8	ANON			80
9	ANON			40
10	ANON			50 (1960)
11	ANON			60-70
12	ANON			9
13	ANON			20
14	ANON			1
15	ANON			3

Age of the slums as per KSCB surveys

Slum id	Slum name	KSCB 1994	KSCB 2008	KSCB 2010
16	ANON			3
17	ANON			
18	ANON			40
19	ANON			1
20	ANON		30 (1980)	35
21	ANON			35
22	ANON			
23	ANON			7
24	ANON			9
25	ANON			35
26	ANON			17
27	ANON			20
28	ANON	18		35
29	ANON		35 (1975)	50
30	ANON	80	48 (1962)	80
31	ANON			40
32	ANON	43		60
33	ANON			48
34	ANON	13		30
35	ANON			50-60
36	ANON			25

The age of the slums was corroborated based on these multiple versions, and a final version is presented in Table 4.6.

Table 4.6: Age of the slums, final version

Slum id	Slum name	Age as of 2010
1	ANON	40
2	ANON	43
3	ANON	30
4	ANON	30-35
5	ANON	20
6	ANON	70
7	ANON	28
8	ANON	50
9	ANON	40
10	ANON	30

Age of the slums, final version

Slum id	Slum name	Age
11	ANON	65
12	ANON	70
13	ANON	25
14	ANON	25
15	ANON	50
16	ANON	30
17	ANON	80
18	ANON	30
19	ANON	Nomadic slum
20	ANON	23
21	ANON	45
22	ANON	40
23	ANON	40
24	ANON	65
25	ANON	35
26	ANON	20
27	ANON	20
28	ANON	35
29	ANON	60
30	ANON	50
31	ANON	35
32	ANON	40
33	ANON	-
34	ANON	50
35	ANON	60
36	ANON	Nomadic slum

NOTE: The slum names have been anonymised ("ANON") to protect the identity of slum dwellers.

5 | Issues and Resolution

While the dataset has undergone multiple stages of clean-up, both before and after entry into the relational database, due to the highly disaggregated nature of the data that was collected, a portion of the data still contains a few issues.

Table 5.1 lists the issues and their resolution within this dataset.

Table 5.1: Issues with the current dataset

Sl.no	Issue	Resolution
1	The caste details of certain subcastes are not ascertained accurately due to unavailability of data.	Partially resolved in the database. While the subcaste data is accurate, the caste data for subcastes is not clear:<br>*Acharya, ADC, Ajay katta, Al-Dis, Almat Gowdaru, Arjunar, Bagigaru, Batteneyuvavaru, Beig, Bhajanthi, Bindcose, Bogaru, Budagi jana, CSI, Dakkani, Devana shetaru, Dhobrikyasi, Dhoriganu, Ganyar, Garuda Bailpathar, Gobari, Gosayi dungrigara, Hajamaru, Hallelujah, Janaru, Kabari yaru, Kaber, Kacheer, Kalaga pata, Kaliny, Karaga Ethuvavaru, Karaga Theyuvavaru, Khan, Kongaru, Kurubachu, Mangarudi, Manne, Missionary, Mohammed, Mola hodeyuvavaru, Mulla, Paraki, Patan, Pillilar, Pinjar, Poper, Protestant, Pentacost, Roman Catholic, Sheik, Shettiar, Shekanth, Subdeal, Sunni, Syed, Uppu shetru, Valmiki Nayakara, Vodayar, Vysya*
2	Ration card data from the surveys was inaccurate as the respondents reported “Pink” and “Red” ration cards.	**Resolved in the database.** After a detailed review of the types of ration cards, the final list of types of ration cards was created and is currently in use in the database.
3	Slum with slum_id = 7 in the database is Thubarahalli. While data was collected from this slum during the survey, during the data clean-up process, it was ascertained to be unreliable and inaccurate.	**Not resolved in the database.** This is to be excluded from all analyses.
4	The age field contains 99 as the age of some people.	**Resolved in the database.** The age was made NULL.

Issues with the current dataset

Sl.no	Issue	Resolution
5	Occupations reported by the respondents were categorised into twelve categories. However, some occupations could not be categorised as they did not fall under any of these categories.	**Resolved in the database.** These occupations are to be excluded from analyses of occupation data.
6	The travel cost for the people whose only mode of travel was walking, was found to be non-zero. This was made 0 as walking does not have an associated travel cost.	**Resolved in the database.** Travel cost for people whose mode of travel is only walking is made NULL.
7	People who have reported their occupation as “Driver” (tempo, car, autorickshaw) have reported their travel cost, time and distance for the duration of their work-day. Since drivers have constant access to a mode of travel, considering this data for analysis might skew the results.	**Not resolved in the database**. Data related to drivers are to be analysed separately. Travel identifiers (travel_id) of these people are *1, 69, 74, 80, 87, 99, 108, 144, 208, 212, 218, 234, 244, 245, 249, 259, 261, 272, 280, 289, 299, 316, 434, 451, 517, 563, 566, 574, 584, 595, 609, 614, 628, 725, 800, 803, 836, 837, 838, 839, 840, 889, 898, 902, 903, 966, 979, 1096, 1265, 1267, 1293, 1332, 1372, 1375, 1378, 1414, 1536, 1564, 1587, 1588, 1602, 1609, 1651, 1709, 1714, 1788, 1825, 1894, 1896, 1938, 1940, 1943, 2089, 2104, 2180, 2201, 2202, 2314, 2376, 2483, 2558, 2666, 2670, 2689, 2702, 2715, 2792, 2960, 3317, 3343, 3399, 3482, 3510, 3582, 3626, 3668*
8	During data clean-up process, data about travel to household purposes was ascertained to be inaccurate and unreliable.	**Not resolved in the database.** This is to be excluded from all analyses. All rows with type_of_travel=3 are data relating to travel to household purposes.
9	Some people had reported daily costs of travel, with the mode of transport being a bus.	**Resolved in the database.** These costs have been multiplied by 26 to represent monthly costs.

Issues with the current dataset

Sl.no	Issue	Resolution
10	Migration data needs further verification before use. Additionally, the responses to the question of where the individuals migrated from is unreliable and inaccurate.	**Not resolved in the database.** Certain fields such as 37_location_name require further verification. However, state and district data, and the year of migration is available for use, although it is advised not to use the migration data until full clean-up is complete.
11	The expenditure on water per pot is reported for only four slums. Additionally, the costs reported from Gangodanahalli are too high, and are most likely outliers.	**Not resolved in the database.** These costs are to be excluded from analyses. For expendiure related to water, the table 194_207_Expenditure is to be used.
12	Water expenditure was collected on a per-pot, per-month and per-litre basis. This was ascertained to be very inaccurate and unreliable.	**Not resolved in the database.** For expenditure related to water, the table 194_207_Expenditure is to be used.
13	Respondents from Gajendranagar have reported the material of the wall as Mud/Unburnt brick, which has resulted in their housing being classified as Kutcha. The material of the wall is brick, thus making the housing semi-pucca.	**Not resolved in the database.** For purposes of analyses, Gajendranagar can be considered to have semi-pucca housing. Household identifiers: *296, 297, 298, 299, 300, 301, 302, 303, 304, 305, 306, 307, 308, 309, 310, 311, 312, 313, 314, 315*
14	The kitchen type for households in Swagath slum is reported as âĂIJKitchen as part of room in the houseâĂİ while in reality all households in Swagath have a separate kitchen	**Not resolved in the database.** For purposes of analyses, households in Swagath slum can be considered to have their own separate kitchens. Household identifiers: *325, 326, 327, 328, 329, 330, 331, 332, 333, 334, 335, 336, 337, 338, 339, 340, 341, 342, 343, 344*

Issues with the current dataset

Sl.no	Issue	Resolution
15	Certain households have not reported any income.	**Partially resolved in the database.** These households are to be excluded from analyses of income, expenditure and family categorisation. They have not been assigned a family category by earning potential. Household identifiers: *11, 54, 75, 110, 160, 187, 199, 203, 206, 277, 279, 291, 344, 369, 477, 534, 576, 622, 634, 657, 687, 717, 811, 837, 879, 887, 893, 927, 965, 982*
16	Some people had reported weekly wages instead of monthly	**Resolved in the database.** This was multipled by 4 to represent monthly costs.
17	One individual has reported an income of Rs 30000	**Not resolved in the database.** This has a high probability of being an outlier as it is the only response which is greater than 18000. This is to be excluded from analyses.
18	Households have reported multiple reasons for sending/ receiving remittances. For instance, one household has reported that they received a certain amount for marriage and construction purposes.	**Not resolved in the database.** These are to be excluded from analyses as it is not possible to identify the split of the amount according to the reason mentioned.
19	A five-year old was reported to earn Rs. 5000.	**Resolved in the database.** This was removed from the dataset
20	The data relating to assets is not cleaned. The fields for year of purchase and cost are interchanged in the database. Also, the codes for assets need to be verified before use.	**Not resolved in the database.** However, this has undergone multiple rounds of cleaning before entry into the database.

References

Elmasri, R., & Navathe, S. (2008). *Fundamentals of database systems, 5/e*. Pearson Education India.

Mitra, A., & Tsujita, Y. (2006). Migration and well-being at the lower echelons of the economy: A study of delhi slums.

NSSO. (2001). *Concepts and definitions used in the nss.* National Sample Survey Organisation, Ministry of Statistics & Programme Implementation, Government of India.

Ramakrishnan, R., & Gehrke, J. (2000). *Database management systems, 2nd edition*. Osborne/McGraw-Hill.

Appendices

A | Ration Card types

Ration cards are provided by the government to the families who are in need of state support. The Government of Karnataka provides the following types of ration cards:

1. Akshaya Scheme (Yellow card)
2. Antyodhaya Anna Yojane
3. APL card

A.1 Akshaya Scheme (Yellow Card)

This card is provided for the weaker sections, backward class families and for senior citizens. For holders of this card, food grains(rice, wheat, sugar) and kerosine are provided free/at a subsidised rate through government ration depoys, with the help of the central government.

The qualifications for this card are:

1. The annual income in urban areas should be less than Rs. 17,000
2. The applicant must be a resident of Karnataka
3. The applicant must be a resident of slums, non wagers, agricultural workers, who has benifits of ashraya scheme, the families who have been living in slums from a long time.

The following are grounds for disqualification

1. Families with permanent landline telephone connections
2. Any government/private labourer whose income is more than Rs. 1000
3. Families which own a own diesel/ petrol vehicles (except mopeds such as TVS 50/ Luna).
4. Families which have outstanding loans of 1 lakh or more

Table A.1 describes the facilities available through this card.

Table A.1: Facilities available through a Yellow Card

Items	Ration in kgs	Rate in Rs
Rice	4(20)	3
Wheat	3	5
Sugar	1	13.5
Kerosene	7	15.5

A.2 Anthyodaya Anna Yojane

This card is for people who are below the poverty line, and for the families who can't afford sufficient meals even for a day. Widows, handicaft workers, weaker sections, mentally retarded, schedule caste, schedule tribes, agricultural labourers and tribal groups are eligible for this card. Table A.2 provides the facilities available through this card.

Table A.2: Facilities available through an Anthyodaya Anna Yojane card

Items	Ration in kgs	Rate in Rs
Rice	29	3
Wheat	4	2
Sugar	1	13.5
Kerosene	7	15.5

A.3 APL card

The Above Poverty Line ration card is for families who do not qualify for the fixed qualification criteria of the Akshaya, Annapurna, Anthyodaya ration cards. Table A.3 describes the facilities available through this card.

Table A.3: Facilities available through an Anthyodaya Anna Yojane card

Items	Ration in kgs	Rate in Rs
Rice	0	0
Wheat	0	0
Sugar	0	0
Kerosene	7	15.5

B | EER Diagram Guide

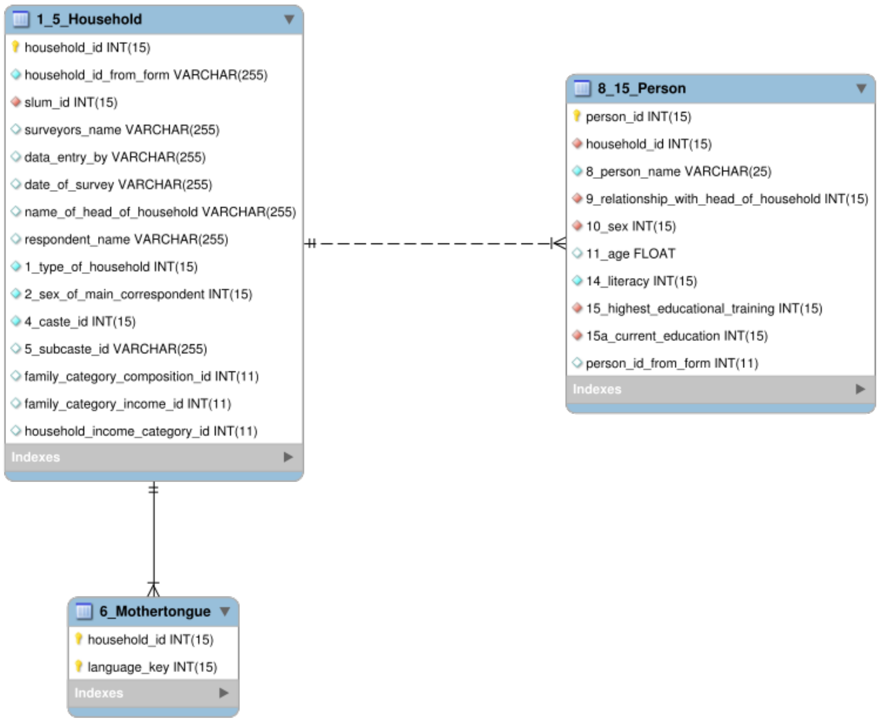

Figure B.1: Sample EER diagram

Figure B.1 shows a sample EER diagram between the tables 1_5_Household, 6_MotherTongue and 8_15_Person.

Fields in the tables

Primary key

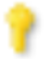

The key symbol in tables indicates its primary key. Most tables have one column as a primary key, while some tables have a combination of two columns as their primary key (a compound key). In the above example, household_id is the primary key of the table 1_5_Household.

Foreign key

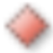 Red diamond symbols indicate that the field is a foreign key. In the above example, "slum_id" is a foreign key in 1_5_Household.

Null fields

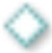 Diamond symbols with only an outline indicate that the field is **nullable**. In the above example, "surveyors_name", "data_entry_by" and "date_of_survey" are some of the nullable fields in 1_5_Household.

Not-null fields

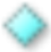 Blue diamond symbols indicate that the field is **not nullable**. In the above example, "1_type_of_household" is a compulsory fields, and cannot be NULL.

Relationships

Relationships in the schema for this data are one-to-many relationships. These relationships are indicated by the following symbology:

Dotted lines

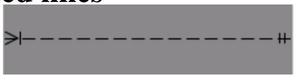

Dotted lines indicate a non-identiyfing relationship: when the foreign key from the referenced table is not a primary key in the referencing table. In the above example, household_id in 8_15_Person is a foreign key from the table 1_5_Household and is **not a primary key in 8_15_Person**

Non dotted lines

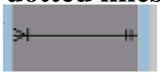

Non-dotted lines indicate identiyfing relationships: when the foreign key from the referenced table **is a key in the referencing table**. In the above example, household_id in the table 6_MotherTongue is a foreign key from the table 1_5_Household and is a primary key in 6_MotherTongue.

C Sample SQL Queries

This chapter presents a few example queries, whose outputs can be used to create contingency tables in any statistical software.

```
SELECT income_category_name,educational_level_name,count(*)

FROM `193_Income`,8_15_Person,HighestEducationalLevel,
      IncomeCategory, 1_5_Household

WHERE 193_Income.person_id=8_15_Person.person_id
AND 8_15_Person.household_id=1_5_Household.household_id
AND
8_15_Person.15_highest_educational_training=
HighestEducationalLevel.educational_level_id
AND 193_Income.income_category_id=IncomeCategory.income_category_id
AND HighestEducationalLevel.educational_level_id NOT IN (10,11)
AND slum_id NOT IN (7)

GROUP BY IncomeCategory.income_category_id,
HighestEducationalLevel.educational_level_id

ORDER BY IncomeCategory.income_category_id
```

Listing 1: Income category and Educational levels of individuals

The listing “Income category and Educational levels of individuals” will output the count of people (N) first grouped by their income category, then grouped by their highest educational training. The output from this query can be used to create a crosstabulation/ contingency table between income categories of people and their highest educational training.

The listing “Occupation and Occupation benefits” will output the count of people (N) first grouped by their occupation category, then grouped by the benefits they get from their occupation. The output from this query can be used to create a crosstabulation/ contingency table between occupation categories and the benefits these occupations provide.

The listing “Travel mode and Slums” will output the count of people (N) first grouped by the slum they live in, then grouped by the travel modes they use. The output from this query can be used to create a crosstabulation/ contingency table between slums and the travel modes their residents use.

```sql
SELECT occupation_category_name, benefit_details, COUNT( * )
FROM 17_23_CurrentOccupation, OccupationCategory, 24_OccBenefit,
      OccupationBenefits, 1_5_Household, 8_15_Person
WHERE 17_23_CurrentOccupation.current_occupation_id =
24_OccBenefit.current_occupation_id
AND 24_OccBenefit.benefit_id = OccupationBenefits.benefit_id
AND 17_23_CurrentOccupation.`occupation_category_id` =
OccupationCategory.`occupation_category_id`
AND 17_23_CurrentOccupation.occupation_category_id NOT IN (13)
AND 17_23_CurrentOccupation.17_person_id = 8_15_Person.person_id
AND 8_15_Person.household_id = 1_5_Household.household_id
AND 1_5_Household.slum_id NOT IN (7)
GROUP BY OccupationCategory.occupation_category_id,
OccupationBenefits.benefit_id
```

Listing 2: Occupation and Occupation benefits

```sql
SELECT slum_name, travel_mode_name, count(
DISTINCT 57_71_Travel.person_id )

FROM 57_71_Travel, 59_71_ModesOfTravel,
    8_15_Person, 1_5_Household, Slum,
    TravelMode

WHERE 57_71_Travel.travel_id = 59_71_ModesOfTravel.travel_id
AND 57_71_Travel.person_id = 8_15_Person.person_id
AND 8_15_Person.household_id = 1_5_Household.household_id
AND 1_5_Household.slum_id = Slum.slum_id
AND 59_64_69_mode_id = TravelMode.travel_mode_id

GROUP BY slum_name, travel_mode_name
```

Listing 3: Travel mode and Slums

D | Variables

The survey had 242 questions spread across the following themes:

Demographics Data on the household details, individual details, and other demographic information.

Employment Data about people's occupation, past occupations, seasonal occupation, employment benefits and self employment.

Migration Data on Migration details of individuals and families in the slums.

Mobility Data on individuals' travel time, cost and distance to work and education, their opinions of the Metro, issues relating to cycling and walking.

Water Data on sources of water used in the summer and other seasons, water availability, distance to water sources, waiting time and expenditure on water.

Housing Data on material of houshold roof, wall and floor, kitchen details, bathroom and toilet details, housing finance details and household size details.

Loans and Remittances Data on loans received and loans provided to other households.

Assets Data on what asset the households own, which year it was purchased, and its price at the time of purchase.

Aspirations, Issues, Agencies and Benefits Qualitative data on the aspirations of the people, their current and most pressing issues and how they resolve them or want to resolve them.

Tables D.1 to D.11 provide a complete list of all the questions asked in the survey, and their mapping on to the database. Chapter 3 describes the database design in detail.

Table D.1: Household and demographics

Q.no	Question from survey instrument	Database Table Name	Column Name
1	Type of household	1_5_Household	1_type_of_household
2	Sex of main respondent	1_5_Household	2_sex_of_main_correspondent
3	Religion	3_Religion	religion_key
4	Caste	1_5_Household	4_caste_id
5	Sub-Caste	1_5_Household	5_subcaste_id
6	Mother tongue	6_MotherTongue, 6_otherMotherTongue	language_key, 6_other_mother_tongue
7	Person Id	8_15_Person	person_id_from_form
9	Relationship with Head of Household	8_15_Person	9_relationship_with_head_of_household
10	Sex	8_15_Person	10_sex
11	Age	8_15_Person	11_age
12	Marital Status	12_13_MaritalDetails	12_marital_status
13	Age at Marriage	12_13_MaritalDetails	13_age_at_marriage
14	Literacy: able to read and write?	8_15_Person	14_literacy
15	Highest educational level completed	8_15_Person	15_highest_educational_training
15a	Currently attending?	8_15_Person	15a_current_education
16	Ration Card	16_RationCardExists	16_type_of_ration_card

Table D.2: Employment

Q.no	Question from survey instrument	Database Table Name	Column Name
17	Person Id	17_23_Occupation	17_person_id
18	Occupation	17_23_Occupation	18_occupation
19	Type of Employer	17_23_Occupation	19_employer_type
20	Seasonality worker.Yes/No	20_21_SeasonalOccupation	Row exists if the answer is Yes
21	Job during off season	20_21_SeasonalOccupation	21_occupation_id

Employment

Q.no	Question from survey instrument	Database Table Name	Column Name
22	How many hours do you work per week?	17_23_Occupation	22_no_hours_per_week
23	No. of Working months during last year	17_23_Occupation	23_no_months_employed_last_year
24	Do you get any work benefits?	24_OccBenefits	benefit_id
25	Do you have any changes in the occupation. What	25_27_PreviousOccupation	25_occupation_name
26	When was the occupation changed	25_27_PreviousOccupation	26_year_of_switch
27	Why was the occupation changed	27_reasonForJobSwitch	27_reason_key
28	What would improve your livelihood Opportunities	28_ImproveWorkOpps	28_improvement
29	Self Employment	29_32_33_SelfEmployment	29_details
30	Instruments/Equipment used for employment	29_32_33_SelfEmployment	30_toolsUsed
31	Instruments/equipment owned /rented	29_32_33_SelfEmployment	31_toolsOwned
32	Capital Invested for employment Initially	29_32_33_SelfEmployment	32_initial_capital
33	Capital Invested for employment , recurring	29_32_33_SelfEmployment	33_periodic_investment
34	Source of Capital	34_selfEmpSourceOfCapital	34_source_key
35	Place of Employment	35_placeOfSelfEmp	35_place_key

Table D.3: Migration

Q.no	Question from survey instrument	Database Table Name	Column Name
36	Person Id	36_44_Migration	36_person_id
38	Name of the state of last previous residence.	36_44_Migration	38_state

Migration

Q.no	Question from survey instrument	Database Table Name	Column Name
39	Name of the district/town of last previous residence.	36_44_Migration	39_district
40	Urban/Rural	36_44_Migration	40_class_of_area
41	IF Urban Slum/non slum	36_44_Migration	41_slum_or_no_slum
42	Distance from present location in kms	36_44_Migration	42_distance_from_current_location
43	Why did you move	43_reasonForMigration	43_reason_key
44	When Did you move?	36_44_Migration	44_year_of_migration
45	Why did you choose to live in this slum?	45_ReasonForMigratingIntoCurrentSlum	45_reason
46	Would you prefer to live in different slum. Yes/No?	46_47_PreferToStayInDifferentSlum	46_yes_or_no
47	Why do you prefer to live in different slum?	46_47_PreferToStayInDifferentSlum	reason_for_preference
48	Would you prefer to live in a different place. Yes/No	48_49_PreferenceOfDifferentPlace	48_yes_or_no
49	Why do you prefer to live in a different place?	48_49_PreferenceOfDifferentPlace	reason
50	Do you know anyone who has moved out of your slum. Yes/No	50_52_MovedOutOfSlum	50_yes_or_no
51	Where did they have move?	50_52_MovedOutOfSlum	51_where_to
52	Why did they move?	50_52_MovedOutOfSlum	52_why
53	Did you bring anyone outside your HH to this slum to live. Yes/No	53_55_MovedIntoSlum	53_yes_or_no
54	If Yes, Relationship	53_55_MovedIntoSlum	54_relationship_with_person
55	Why did you bring?	53_55_MovedIntoSlum	55_why

Table D.4: Travel

Q.no	Question from survey instrument	Database Table Name	Column Name
57	Travel to Workplace distance in Kms	57_71_Travel	57_62_and_67_distance
58	Travel to Workplace travel Time	57_71_Travel	58_63_and_68_travel_time
59	Travel to Workplace mode of Travel	59_71_ModesOfTravel, 59_64_69_OtherTravelMode	59_64_69_mode_id, 59_64_69_other_travel_mode
60	Travel to Workplace why this Mode?	60_65_70_WhyThisMode, 60_65_70_OtherReason	60_65_70_why, 60_65_70_other_reason
61	Travel to Workplace cost in rupees	57_71_Travel	61_66_71_cost
62	Travel to Education distance in Kms	57_71_Travel	57_62_and_67_distance
63	Travel to Education travel Time	57_71_Travel	58_63_and_68_travel_time
64	Travel to Education Mode	59_71_ModesOfTravel, 59_64_69_OtherTravelMode	59_64_69_mode_id, 59_64_69_other_travel_mode
65	Travel to Education why this Mode?	60_65_70_WhyThisMode, 60_65_70_OtherReason	60_65_70_why, 60_65_70_other_reason
66	Travel to Education cost in Rupees	57_71_Travel	61_66_71_cost
67	Travel for Household Purchases Distance in Kms	57_71_Travel	57_62_and_67_distance
68	Travel for Household Purchases Travel Time	57_71_Travel	58_63_and_68_travel_time
69	Travel for Household Purchases mode of Travel	59_71_ModesOfTravel, 59_64_69_OtherTravelMode	59_64_69_mode_id, 59_64_69_other_travel_mode
70	Travel for Household Purchases why this Mode?	60_65_70_WhyThisMode, 60_65_70_OtherReason	60_65_70_why, 60_65_70_other_reason
71	Travel for Household Purchases cost in Rupees	57_71_Travel	61_66_71_cost
72	Changes in Mode From	72_74_ChangeInTravelMode	72_from_key
73	Changes in Mode To	72_74_ChangeInTravelMode	73_to_key
74	When was the mode changed?	72_74_ChangeInTravelMode, 74_OtherModeOfTravel	74_when, 74_other_mode
75	Why was the mode changed?	75_ReasonForChangeInMode, 75_OtherReasonForChangeInMode	75_why, 75_other_reason

Travel

Q.no	Question from survey instrument	Database Table Name	Column Name
76	How do you get information on transport options?	76_MethodOfGettingTransport--Information, 76_OtherMethodOfGetting--TransportInformation	76_method_key, 76_other_method
78	Quality of bus Service, Do you have any issues	78_IssuesWithBus	78_issue_key
79	What would you change to improve bus service ?	77_79_BusIssue	79_changes
80	Have you heard about the Metro Yes/No	80_82_Metro	80_yesOrNo
81	Would you use it?	80_82_Metro	81_would_you_use_it
82	Why Would you use it?	80_82_Metro	82_why
83	Do you Use a Cycle? Yes/No	83_85_Cycle	83_yesOrNo
84	If yes rent own or borrowed	83_85_Cycle	84_rented_or_borrowed
85	Nearest cycle shop for rentals?	83_85_Cycle, 85_OtherCycleRentDistance	85_nearest_shop_rental, 85_distance
87	Do you face any issues in walking ?	86_87_IssuesInWalking, 87_OtherIssuesInWalking	87_issue, 87_other_issue
88	What can be improved to make walking a better experience	88_BetterWalkingExperience	88_walking_issues
89	Do you face any issues in cycling?	89_CyclingIssues, 89_otherCyclingIssues	89_issue_key, 89_other_issue
90	What can be done to make cycling a better experience?	90_ImproveCycling	90_issue

Table D.5: Water

Q.no	Question from survey instrument	Database Table Name	Column Name
91	Water souces used in Other Seasons	91_92_WaterSourcesUsed	91_92_season_key
92	Water souces used in Summer	91_92_WaterSourcesUsed	91_92_season_key
93	Number of households sharing this water source?	93_94_WaterSources	93_no_of_households_sharing_source
94	Service provider	93_94_WaterSources, 94_OtherServiceProvider	94_service_provider_key, 94_other_service_provider
95	Days per week that water is available from this source in Other Seasons	95_96_WaterAvailabilityDaysPerWeek	95_96_water_availability_days_per_week
96	Days per week that water is available from this source in Summer	95_96_WaterAvailabilityDaysPerWeek	95_96_water_availability_days_per_week
97	Hours per day that water is available from this source in Other Seasons	97_98_WaterAvailabilityHoursPerWeek	97_98_hours_availability_per_day
98	Hours per day that water is available from this source in Summer	97_98_WaterAvailabilityHoursPerWeek	97_98_hours_availability_per_day
99	HouseholdâĂŹs water source(s)	99_101_CostOfWaterProcurement	99_water_source
100	When was this water connection established ?	99_101_CostOfWaterProcurement	100_when_the_connection_was_established
101	What was the cost to the household of establishing the connection (Rs)?	99_101_CostOfWaterProcurement	101_cost_to_the_household
102	Do you pay per container or a fixed amount each month in Other Seasons	102_103_waterExpenditureOnPot	102_103_expenditure
103	Do you pay per container or a fixed amount each month in Summer	102_103_waterExpenditureOnPot	102_103_expenditure
104	Price per liter? Other Seasons	104_105_waterExpenditurePerLiter	104_105_season_key, expenditure
105	Price per liter? Summer (Calculate based on price per container and container size)	104_105_waterExpenditurePerLiter	104_105_season_key, expenditure
106	what is the fixed price or access fee per month in Other Seasons	106_107_monthlyWaterExpenditure	106_107_season_key, expenditure
107	what is the fixed price or access fee per month (Rs.) in Summer	106_107_monthlyWaterExpenditure	106_107_season_key, expenditure

Water

Q.no	Question from survey instrument	Database Table Name	Column Name
108	Who is collecting the Water charges?	108_AgencyCollectingWaterCharges	108_agency_collecting_water_charges
109	How far is the water source from your house in Other Seasons(meters)	109_110_distanceOfWaterSource	109_110_season_key, distance
110	How far is the water source from your house in Summer	109_110_distanceOfWaterSource	109_110_season_key, distance
111	How long does it take to get to the water source in Other Seasons	111_112_timeToWaterSource	111_112_season_key, time
112	How long does it take to get to the water source in Summer	111_112_timeToWaterSource	111_112_season_key, time
113	How long is the waiting and filling time per collection? In other seasons	113_114_waitingTimeAtWaterSource	113_114_season_key, waiting_time
114	How long is the waiting and filling time per collection? Summer	113_114_waitingTimeAtWaterSource	113_114_season_key, waiting_time
115	How many household members typically go to collect water in each trip in Other Seasons	115_116_peopleGoingToCollectingWater	115_116_season_key, number_of_people
116	How many household members typically go to collect water in each trip in Summer	115_116_peopleGoingToCollectingWater	115_116_season_key, number_of_people
117	How often do household members go to this source to get water each week in Other Seasons	117_118_frequencyOfGoing--ToWaterSource	117_118_season_key, frequency
118	How often do household members go to this source to get water each week in Summer	117_118_frequencyOfGoing--ToWaterSource	117_118_season_key, frequency
119	How much water do the household members collect per trip in total? A	119_124_RoundTripWaterCollection	119_121_volume_of_water
120	How much water do the household members collect per trip in total? B	119_124_RoundTripWaterCollection	119_121_volume_of_water
121	How much water do the household members collect per trip in total? C	119_124_RoundTripWaterCollection	119_121_volume_of_water

Water

Q.no	Question from survey instrument	Database Table Name	Column Name
122	Who typically procures water from this source? A	119_124_RoundTripWaterCollection	122_124_person
123	Who typically procures water from this source? B	119_124_RoundTripWaterCollection	122_124_person
124	Who typically procures water from this source? C	119_124_RoundTripWaterCollection	122_124_person
	Is water from this source used for drinking in Other Seasons?	124_125_isWaterFromSource--UsedForDrinking	is_water_used_for_drinking, 124_125_season_key
125	Is water from this source used for drinking in Summer?	124_125_isWaterFromSource--UsedForDrinking	is_water_used_for_drinking, 124_125_season_key
126	Is water from this source used for cooking in Other Seasons	126_127_isWaterUsedForCooking	is_water_used_for_cooking, 124_125_season_key
127	Is water from this source used for cooking in Summer	126_127_isWaterUsedForCooking	is_water_used_for_cooking, 124_125_season_key
128	During a typical week, how often do household members use water from each source in Other Seasons	128_129_isWaterUsedForBathing	is_water_used_for_bathing, 124_125_season_key
129	During a typical week, how often do household members use water from each source in Summer	128_129_isWaterUsedForBathing	is_water_used_for_bathing, 124_125_season_key
130	Treating Water at Home?	131_134_WaterTreatment	
131	Method used for treating water	131_134_WaterTreatment	purification_method_key
132	Year of purchase of water treating machine.	131_134_WaterTreatment	132_year_of_purchase
133	Purchase Price (Rs) of the water treating machine	131_134_WaterTreatment	133_purchase_price
134	Annual cost of operation/mantainence(Rs)	131_134_WaterTreatment	134_annual_maintainance_cost
135	Household has water meter?	135_WaterMeterExistence	135_household_has_water_meter
136	Average monthly water consumption (in KL)	136_WaterConsumption	136_consumption

Water

Q.no	Question from survey instrument	Database Table Name	Column Name
137	Water bill is received regularly?	137_IsWaterBillRegular	137_is_water_bill_regular
138	How many months does the bill cover?	138_MonthsCoveredByWaterBil	138_months_covered
139	Is the Bill accurate?	‘139_IsWaterBillAccurate	139_is_water_bill_accurate
140	Can you afford to pay the bill?	140_IsWaterBillAffordable	140_IsWaterBillAffordable
141	Water bill is based on	141_WaterBillBasedOn	141_based_on_key
142	Is Water provided to neighbours	142_IsWaterProvidedByNeighbours	142_is_water_provided_by_neighbours
143	Monthly earning from selling to neighbours	143_MonthlyEarningFromSelling--ToNeighbours	143_monthly_earning

Table D.6: Housing

Q.no	Question from survey instrument	Database Table Name	Column Name
144	Size of the house plot	144_SizeOfHousePlot	144_plot_size
145	Area occupied by the household for living space	145_AreaOccupiedForLiving	145_area_occupied_for_living
146	Predominant material of floors	146_FloorMaterial	146_floor_material_key
147	Predominant material of walls	147_WallMaterial	147_wall_material_key
148	Predominant material of roof	148_RoofMaterial	148_roof_material_key
149	Use of the dwelling unit	149_UseOfDwellingHouse	149_use_of_dwelling_house
150	Number of rooms in dwelling unit Excl. Kitchen and bathrooms	150_NumberOfRoomsInHouse	150_number_of_rooms
151	Number of rooms occupied by household	151_NumberOfRoomsOccupied--ByHousehold	151_number_of_rooms_occupied_bu_household
152	Number of rooms/sq feet used for Home based manufacturing or common purposes	152_NoOfRoomsOrSqft	152_number_of_rooms_or_sqft
153	Type of Bath rooms	153_BathroomType	153_bathroom_type_key
154	What changes do you think are required to improve the bathroom ?	154_BathroomImprovements	154_bathroom_improvements

Housing

Q.no	Question from survey instrument	Database Table Name	Column Name
155	Type of kitchen	155_KitchenType	155_kitchen_type_key
156	Do you have a toilet at home?	156_ToiletExistence	156_toilet_existence
157	Type of Toilet Facilities	157_ToiletFacilities	157_toilet_facilities_key
158	Where are the wastes from toilet discharged	158_ToiletWasteDisposal	158_toilet_waste_disposal_key
159	What changes do you think are required to improve the toilet?	159_ToiletImprovements	159_toilet_improvements
160	Type of access to electricity	160_ElectricityAccessType	160_electricity_access_key
161	To whom are electricity charges are paid?	161_ElectricityBillPaidTo	161_electricity_bill_paid_to_key
162	Monthly payment of electricity	162_ElectricityMonthlyBill	162_electricity_monthly_bill_amount
163	Types of cooking fuel	163_TypeOfCookingFuel	163_type_of_cooking_fuel
164	Monthly expenditure on cooking fuel	164_CookingFuelPrice	164_expenditure
165	For leased accommodation: how much was lease payment made to house-owner (Rs.)	165_LeaseAmountIfRentedHouse	165_lease_amount
166	Current monthly rent (Rs.)	166_MonthlyRent	166_monthly_rent
167	Amount of security payment(advance) to the house owner	167_SecurityPaymentToOwner	167_security_payment_amount
168	How much was the monthly rent when dwelling unit was first rented?	168_MonthlyRentWhenUnitWasBuilt	168_monthly_rent_when_unit_was_built
169	Has landlord made improvements to dwelling unit?	169_ImprovementsToDwelling- -UnitByLandlord	169_improvement_key
170	Did your family construct the house?	170_FamilyConstructedHouse	170_family_constructed_house
171	Year that house was constructed	171_YearOfHouseConstruction	171_year_of_house_construction

Table D.7: Housing Finance

Q.no	Question from survey instrument	Database Table Name	Column Name
172	Household has legal title to the dwelling?	172_LegalTitleToDwelling	172_legal_title_to_dwelling

Housing Finance

Q.no	Question from survey instrument	Database Table Name	Column Name
173	Approximate total cost of construction Rs.	173_ApproxTotalConstructionCost	173_approx_total_construction_cost
174	Approximate total cost of land Rs.	174_ApproxTotalCostOfLand	174_approx_total_cost_of_land
175	Year land was acquired	175_YearLandWasAcquired	175_year_land_was_acquired
176	Year home was acquired	176_YearHomeWasAcquired	176_year_home_was_acquired
177	Approximate purchase price	177_ApproxPurchasePrice	177_approx_purchase_price
178	How did your finance your current home (Enter all that apply) Amount	178_181_CurrentHomeFinance	178_amount
179	How did your finance your current home (Enter all that apply) Annual interest rate	178_181_CurrentHomeFinance	179_annual_interest_rate
180	How did your finance your current home (Enter all that apply)Term (year)	178_181_CurrentHomeFinance	180_term_in_years
181	How did your finance your current home (Enter all that apply). Equated monthly instalments (Rs)	178_181_CurrentHomeFinance	181_emi
182	Estimated present market price for a similar uit in this neighbourhood (Rs.)	182_PresentMarketPrice	182_market_price
183	Estimated monthly rental value for a similar unit in this neighbourhood (Rs)	183_EstimatedMonthlyRental- -ForSimilarUnit	183_estimated_monthly_rental
184	Satisfied with current dwelling?	184_SatisfiedWithCurrentDwelling	184_satisfied
185	If no, household wants to	185_IfNotSatisfiedWithDwelling	185_not_satisfied
186	What upgrade would be most important ?	186_MostImportantUpgrade	186_most_important_upgrade
187	Reasons for wanting to move	187_ReasonsForWantingToMove	187_reasons_for_wanting_to_move
188	Planning to move within the next 12 months?	188_PlanningToMoveWithin- -TwelveMonths	188_planning_to_move_key
189	Is your neighbourhood safe If yes, How?	189_IsNeighbourhoodSafe, 189_ReasonNeighbourhoodIsSafe	189_is_neighbourhood_safe, 189_reason

Table D.8: Income and Expenditure

Q.no	Question from survey instrument	Database Table Name	Column Name
190	Income. Person Id	191_192_HouseholdPeopleDetails	person_id
191	House hold members	191_192_HouseholdPeopleDetails	191_person_name
192	Sex of HH member	191_192_HouseholdPeopleDetails	192_sex
193	Income	193_Income	income_category_id
194	How much towards home	194_207_Expenditure	194_home
195	How much towards Food	194_207_Expenditure	195_food
196	How much towards Fuel	194_207_Expenditure	196_fuel
197	How much towards Transport	194_207_Expenditure	197_transportation
198	How much towards Communication	194_207_Expenditure	198_communication
199	How much towards Water	194_207_Expenditure	199_water
200	How much towards Electricity	194_207_Expenditure	200_electricity
201	How much towards Clothing	194_207_Expenditure	201_clothing
202	How much towards Education	194_207_Expenditure	202_education
203	How much towards Medical Care	194_207_Expenditure	203_medication
204	How much towards Housing	194_207_Expenditure	204_housing
205	How much towards Religious Events	194_207_Expenditure	205_religious
206	How much towards Entertainment	194_207_Expenditure	206_entertainment
207	How much towards Others	194_207_Expenditure	207_others

Table D.9: Remittances and Loans

Q.no	Question from survey instrument	Database Table Name	Column Name
208	Do you own any immovable property in your native place	208_OwnImmovablePropertyAt--NativePlace	208_own_immovable_property
209	If yes, what is it?	209_TypeOfImmovableProperty	209_type_of_property
210	Location of Property?(village/town/etc)	210_LocationOfImmovableProperty	210_district_of_property,210_state_of_property

Remittances and Loans

Q.no	Question from survey instrument	Database Table Name	Column Name
211	Money Sent. Any cash assistance provided during the last 12 months by household to a relative or another person not residing in the household	211_CashAssistanceProvided	211_was_cash_assistance_provided
212	Money Sent, For whom did the household send money during the last 12 months	212_216_CashAssistanceProvidedDetails	212_relationship_with_receiver
213	Money Sent, R/U code	212_216_CashAssistanceProvidedDetails	213_urban_or_rural
214	Money Sent, List place (State/District, or Country)	212_216_CashAssistanceProvidedDetails	214_place
215	Money Sent, Purpose of Remittance	212_216_CashAssistanceProvidedDetails	215_reason
216	Amount Sent	212_216_CashAssistanceProvidedDetails	216_amount
217	Money Received, Any cash assistance received during the last 12 months by household to a realtive or another person not residing in the household	217_CashAssistanceReceived	217_was_cash_assistance_received
218	Money Received, From whom did the household received remittances during the last 12 months	218_223_CashAssistanceReceived	218_relationship_with_sender
219	Money Received, R/U code	218_223_CashAssistanceReceived	219_urban_rural
220	Money Received List place (State/District, or Country)	218_223_CashAssistanceReceived	220_place
221	Money Received Form of Remittance	218_223_CashAssistanceReceived	221_loan_or_gift
222	Money Received Purpose of Remittance	218_223_CashAssistanceReceived	222_reason
223	Amount received	218_223_CashAssistanceReceived	223_amount

Table D.10: Household Assets

Q.no	Question from survey instrument	Database Table Name	Column Name
224	Household Assets	224_227_HouseholdAssets, 224_227_vehicleRent	224_asset_key
225	Number	224_227_HouseholdAssets, 224_227_vehicleRent	225_number
226	Year Acquired	224_227_HouseholdAssets, 224_227_vehicleRent	226_year
227	Prince paid or value at time of acquistition (Rs.)	224_227_HouseholdAssets, 224_227_vehicleRent	227_price_at_the_time_of_acquisition

Table D.11: Issues, Agencies and Benefits

Q.no	Question from survey instrument	Database Table Name	Column Name
228	What are the most pressing problems you face?	228_MostPressingProblems	228_most_pressing_problems
229	When you have a problem, how do you try to reslove it ?	229_HowProblemIsResolved	229_how_problem_is_resolved
230	Are you usually able to resolve problems you face?	230_AreYouAbleToResolveProblems	230_are_you_able_to_resolve_problem
231	IF you could change process of problem resolution, What?	231_ChangeProcessOfProblemResolution	231_change_process_of_problem_resolution
232	Do you know of any organisation working for your welfare.	232_OrganisationWorkingForWelfare	232_organisation_key
233	If yes, What activities do they do	233_ActivitiesOfOrganisation	233_activities_of_organisation
234	Have you interacted with	234_235_InteractedWithOrganisation	234_organisation_key
235	If yes, for what?	234_235_InteractedWithOrganisation	235_interaction_about
236	If you are sick, What do you do to get better?	236_IfSickWhatIsDoneToGetBetter	236_WhatIsDoneToGetBetter
237	How far away is a healthcenter?	238_DistanceOfHealthCenter	238_distance

Issues, Agencies and Benefits

Q.no	Question from survey instrument	Database Table Name	Column Name
238	What changes do you want to happen to improve your access to health services?	240_ChangesToHealthService	240_changes
239	In five years what changes, could improve your life	237_ChangesThatCouldImprove--LifeInFiveYears	237_changes
240	What are you doing towards making those changes	239_WhatIsBeingDoneToward--ThoseChanges	239_what_is_being_done
241	What should the government to help make those changes?	241_WhatGovtShouldDo	241_what_govt_should_do
242	Is there anything that you would like to tell us outside of the questions we have asked.	242_AnyOtherDetailsNotAlreadyAsked	242_other_details

Table D.12: Code tables in the database

Sl.no	Referring Table	Referenced Table/ Code Table
1	1_5_Household	Caste
2	1_5_Household	ClassOfSlum
3	1_5_Household	CurrentlyAttending
4	1_5_Household	FamilyCategoryByComposition
5	1_5_Household	FamilyCategoryByEarningPotential
6	1_5_Household	HouseholdIncomeCategory
7	1_5_Household	HouseholdRelationshipCode
8	1_5_Household	HousingType
9	1_5_Household	Slum
10	1_5_Household	TypeOfHousehold
11	3_Religion	Religion
12	6_MotherTongue	Language
13	8_15_Person	Gender
14	8_15_Person	HighestEducationalLevel
15	8_15_Person	IncomeCategory
16	12_13_MaritalDetails	Marital_status
17	16_RationCardExists	RationCardType
18	17_23_CurrentOccupation	EmployerTypeCode
19	17_23_CurrentOccupation	OccupationCategory
20	24_OccBenefit	OccupationBenefits
21	27_reasonForJobSwitch	ReasonsforOccupationSwitch
22	34_selfEmpSourceOfCapital	CapitalSource
23	35_placeOfSelfEmp	PlaceforOccupation
24	36_44_Migration	UrbanRural
25	43_reasonForMigration	MigrationCodes
26	45_ReasonForMigrating-<br>-IntoCurrentSlum	ReasonToMoveIntoSlumCode
27	48_49_PreferenceOfDifferentPlace	ReasonsForWantingToMove
28	50_52_MovedOutOfSlum	MovedOutOfSlumWhereToCodes
29	53_55_MovedIntoSlum	MovedIntoSlumRelationshipCode
30	57_71_Travel	TypeOfTravel
31	59_71_ModesOfTravel	TravelMode
32	59_71_ModesOfTravel	WhyThisMode
33	60_65_70_WhyThisMode	TravelModeReasons
34	75_ReasonForChangeInMode	ReasonOfChangeInTravelMode
35	76_MethodOfGetting-<br>-TransportInformation	MethodOfLearningOfTravel
36	78_IssuesWithBus	BusIIssues
37	83_85_Cycle	CycleRent

Table D.12: Code tables in the database

Sl.no	Referring Table	Referenced Table/ Code Table
38	83_85_Cycle	NearestCycleRental
39	86_87_IssuesInWalking	WalkingIssues
40	89_CyclingIssues	CyclingIssues
41	93_94_WaterSources	WaterServiceProviders
42	93_94_WaterSources	WaterSources
43	130_waterPurification	PurificationMethods
44	135_WaterMeterExistence	WaterMeterStatus
45	146_FlootMaterial	FloorMaterial
46	147_WallMaterial	WallMaterial
47	148_RoofMaterial	RoofMaterial
48	149_UseOfDwellingHouse	DwellingUse
49	153_BathroomType	BathroomType
50	155_KitchenType	KitchenType
51	157_ToiletFacilities	ToiletFacilities
52	158_ToiletWasteDisposal	ToiletWasteDisposal
53	160_ElectricityAccessType	ElectricityAccessType
54	161_ElectricityBillPaidTo	ElectricityBillPaidTo
55	163_TypeOfCookingFuel	CookingFuelType
56	169_ImprovementsToDwelling- -UnitByLandlord	ImprovementOfDwellingUnit
57	170_FamilyConstructedHouse	BuiltOrBought
58	178_181_CurrentHomeFinance	HouseFinanceSource
59	185_IfNotSatisfiedWithDwelling	NotSatisfiedWithHousing
60	186_MostImportantUpgrade	MostImportantUpgrade
61	188_PlanningToMove- -WIthingTwelveMonths	MovingPlans
62	212_216_CashAssistanceProvided	ReasonForSendingOrReceivingMoney
63	212_216_CashAssistanceProvided	RelationshipCodes
64	218_223_CashAssistanceReceived	ReasonForSendingOrReceivingMoney
65	218_223_CashAssistanceReceived	RelationshipCodes
66	224_227_HouseholdAssets	HouseholdAssetsCode
67	232_OrganisationWorkingForWelfare	OrganisationForWelfare
68	234_235_InteractedWithOrganisation	InteractedWithOrganisation
69	236_IfSickWhatIsDoneToGetBetter	IfSickWhatIsDone
70	238_DistanceOfHealthCenter	DistanceOfHealthCenter
71	AgeBinsOfPeople	AgeBinsLiteracy
72	AgeBinsOfPeople	AgeBinsOccupation
